# Electro-optic tuning in composite silicon photonics based on ferroionic 2D materials

**DOI:** 10.1038/s41377-024-01432-2

**Published:** 2024-04-19

**Authors:** Ghada Dushaq, Solomon Serunjogi, Srinivasa R. Tamalampudi, Mahmoud Rasras

**Affiliations:** 1 Department of Electrical and Computer Engineering, New York University Abu Dhabi, P.O. Box 129188, Abu Dhabi, United Arab Emirates; 2 NYU Tandon School of Engineering, New York University, New York, NY USA

**Keywords:** Silicon photonics, Optoelectronic devices and components, Integrated optics

## Abstract

Tunable optical materials are indispensable elements in modern optoelectronics, especially in integrated photonics circuits where precise control over the effective refractive index is essential for diverse applications. Two-dimensional materials like transition metal dichalcogenides (TMDs) and graphene exhibit remarkable optical responses to external stimuli. However, achieving distinctive modulation across short-wave infrared (SWIR) regions while enabling precise phase control at low signal loss within a compact footprint remains an ongoing challenge. In this work, we unveil the robust electro-refractive response of multilayer ferroionic two-dimensional CuCrP_2_S_6_ (CCPS) in the near-infrared wavelength range. By integrating CCPS into silicon photonics (SiPh) microring resonators (MRR), we enhance light-matter interaction and measurement sensitivity to minute phase and absorption variations. Results show that electrically driven Cu ions can tune the effective refractive index on the order of 2.8 × 10^−3^ RIU (refractive index unit) while preserving extinction ratios and resonance linewidth. Notably, these devices exhibit low optical losses and excellent modulation efficiency of 0.25 V.cm with a consistent blue shift in the resonance wavelengths among all devices for either polarity of the applied voltage. These results outperform earlier findings on phase shifters based on TMDs. Furthermore, our study demonstrates distinct variations in electro-optic tuning sensitivity when comparing transverse electric (TE) and transverse magnetic (TM) modes, revealing a polarization-dependent response that paves the way for diverse applications in light manipulation. The combined optoelectronic and ionotronic capabilities of two-terminal CCPS devices present extensive opportunities across several domains. Their potential applications range from phased arrays and optical switching to their use in environmental sensing and metrology, optical imaging systems, and neuromorphic systems in light-sensitive artificial synapses.

## Abstract (web summary)

The integration of transition metal thio/selenophosphates (TPS) like CuInPS and CuCrPS onto Silicon Photonics marks a significant advancement in optical devices. This innovation provides precise control over refractive index, minimizes optical losses, enhances modulation efficiency, and reduces the footprint for next-generation optoelectronics.

## Graphical abstract

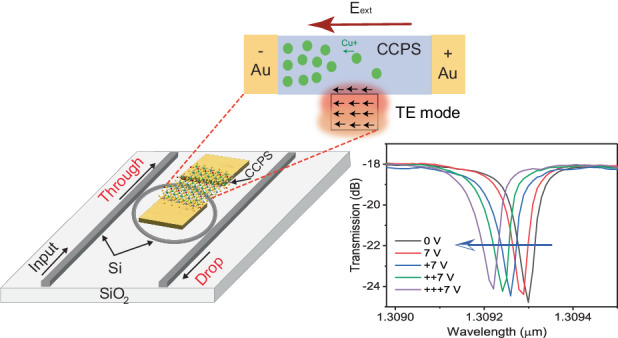

## Introduction

The increasing demands for bandwidth in modern communication networks, optical sensing components, and advanced imaging systems have heightened the need for efficient tunable optical materials designed for precise light modulation^1–4^. Two-dimensional (2D) materials have emerged as outstanding candidates for generating tunable optical components such as electro-optic modulators, switches, and filters, providing numerous possibilities for the precise and effective manipulation of light^5–10^. This attribute is a result of their quantum confinement, which allows for strong interactions between light and matter, leading to a pronounced change in their optical properties in response to external stimuli such as an electric field^11,12^.

A variety of techniques have been developed to tune the optical properties of 2D materials. For instance, light modulation in both the visible and short-wave infrared (SWIR) regions has been successfully demonstrated through the utilization of electrostatic gating in transition metal dichalcogenides (TMDs) when integrated into photonic circuits^7,13–17^. However, in the vicinity of their excitonic resonances, these materials exhibit simultaneous modulation of both the refractive index (Δ*n*) and absorption (Δ*k*). As a result, attaining pure phase modulation presents inherent challenges. Recently, a pure phase modulation based on integrated WS_2_ and MoS_2_ operating in the SWIR (far from their excitonic resonances) has been achieved^7^. The findings demonstrated a high |Δ*n*/Δ*k*| ratio as well as minimal losses during signal transmission. Nevertheless, the realization of phase modulators predicated on capacitive configurations to facilitate electrostatic doping of WS_2_ presents noteworthy challenges predominantly rooted in the intricacies of lithographic processes and precise alignment requirements. This alignment is essential to ensure effective electrostatic doping, as it involves meticulously positioning the WS_2_ in relation to electrodes and other structural elements, which is vital for consistent device performance. Additional approaches, such as the employment of the plasma dispersion effect in silicon-based devices, induce intensity change along with the phase shift^18–20^. Moreover, the use of thermo-optic methods poses significant challenges due to the need for efficient heat dissipation and the maintenance of temperature stability^21^. Hence, identifying new materials with unique modulation properties to control Δn/Δk across SWIR region within a compact footprint is highly desired.

Very recently, there has been a significant surge in interest in 2D ferroionic compounds^22–24^, primarily due to their remarkable characteristics such as moderate bandgaps ranging from 1.3 to 3.5 eV, multiple ferroic orders, and exceptional ionic conductivity^23,25–29^. Notably, Van der Waals (vdW) thio- and selenophosphates such as CuInP_2_S_6_ and CuCrP_2_S_6_ (CCPS) have found application as active dielectrics in electronic devices with two-dimensional or quasi-two-dimensional designs. The overall electronic attributes of these materials are heavily influenced by their bulk ionic conductivity, a critical aspect that can be effectively controlled by manipulating factors such as poling time, polarization, and current direction, all of which are connected to the migration of highly mobile copper (Cu) ions^25,30–33^. Despite the advantageous presence of electrochemically active Cu ions within the lattice and their facile integration into various substrates, current research predominantly focuses on their memristive properties for storage applications and neuromorphic computing^34–37^. However, by combining its multi-ferric order and ionic attributes, CuCrP_2_S_6_ offers a novel pathway to address challenges in achieving pure optical phase modulation in integrated photonics while enabling efficient and versatile light manipulation.

In this study, the electro-optic response of multilayer CCPS integrated on silicon photonics (SiPh) microring resonators (MRR) are actively manipulated at the near-infrared (NIR) wavelengths. Electrically driven Cu ion migration enables precise tuning of the refractive index by ~2.8 × 10^−3^ RIU (refractive index units), while preserving extinction ratios and resonance linewidth. This adjustable electrical conduction arises from the reversible Cu ions accumulation or removal at the metal-semiconductor interface, modulating the contact Schottky barrier height. The inclusion of CCPS on uncladded MRR results in strong light-matter interaction and low optical losses of 2.7 dB cm^−1^. This value is lower than barium titanate devices^38^ and comparable to those of lithium niobate devices^39,40^. Additionally, the devices exhibit excellent modulation efficiency of 0.25 V cm with a consistent blue shift in the resonance wavelengths among all devices. Electro-optic tuning exhibits sensitivity to the light polarization alignment with the CCPS, highlighting its significance. The combined optoelectronic and ionotronic functionalities in the two-terminal CCPS device hold potential for applications, including phased arrays, optical switching, environmental sensing and metrology, optical imaging systems, and neuromorphic systems in light-sensitive artificial synapses.

## Results

### Structural characteristics of the vdW CCPS crystal

**Fig. 1:**
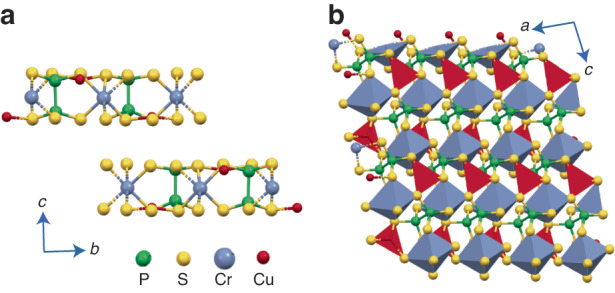
Schematic visualization (ball and stick model). **a** Side view (*bc* plane); **b** 3D representation showing the occupation sites of copper and chromium in CCPS structure

The layered Van der Waals CCPS belongs to the transition metal thio/selenophosphates (TPS) family with a monoclinic crystal structure (Pc space group)^26,41^. Figure 1 shows the side view (bc plane) and a 3D schematic (ac plane) of the material structure. The monolayer is composed of a sulfur framework in which octahedral cages are filled by Cu and chromium (Cr) ions as well as P-P pairs. The Cu ions occupy the upper (Cu1) and lower (Cu2) positions alternately, resulting in an antiferroelectric (AFE) state at low temperatures. Within the crystal structure, triangular networks are formed by quasi-trigonal CuS_3_, octahedral CrS_6_, and P_2_S_6_ units^26,28,42,43^. The Cr ions and the P-P pairs are almost centered within a layer, while the Cu ions are off-centered^44^. Interestingly, the Cu ions become mobile under an external electric field, and they exhibit in-plane and out-of-plane (across the van der Waals gap) movements, which is the primary cause of the ionic conductivity in this layered material as will be discussed in the subsequent sections of the paper.

**Fig. 2:**
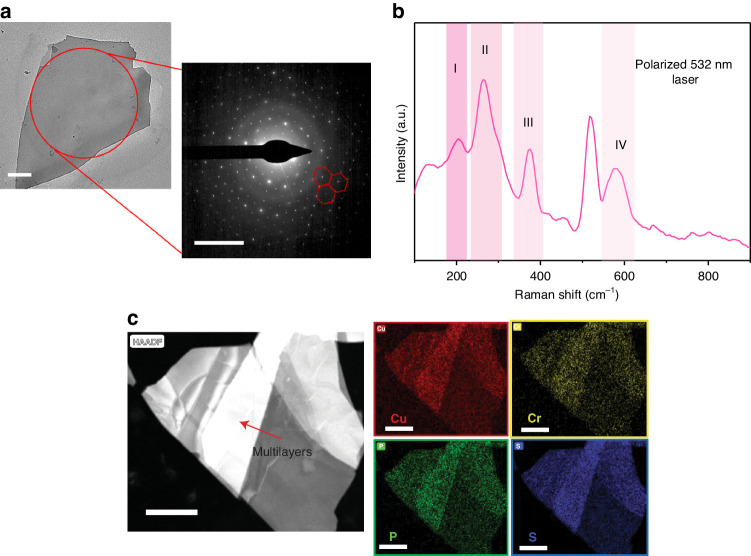
Structural characteristics of CCPS. **a** TEM image along with SADP, the red dashed hexagon is highlighted to guide the eye into the structure, **b** Raman spectra collected using a polarized 532 nm laser, **c** Quantitative EDX mapping of CCPS. The bar readings for (**a**, **c**) are: 500  nm, 10  nm^−1^ and 1 µm, respectively

To quantify the crystal structure, the CCPS flakes are directly exfoliated from their bulk crystals using a mechanical exfoliation technique. To ascertain a good crystalline structure and homogeneity of the layered CCPS, transmission electron microscopy (TEM) testing was carried out. A low-resolution TEM image of an exfoliated flake is shown in Fig. 2a (a high-resolution image is shown in Fig. S1). Additionally, the selected area diffraction pattern (SADP) along the c-axis of the CCPS verified the monoclinic nature and the high consistency of the crystals (see Fig. 2a)^26^. Raman spectra collected from CCPS flakes in the backscattered geometry are shown in Fig. 2b. The four active vibrational modes at room temperature are labeled as I-IV. A comparison between our measured Raman peaks and those reported in the literature reveals that peak I corresponds to in-plane vibrational mode (E_g_), while all other peaks correspond to out-of-plane mode (A_g_)^45^. Quantitative elemental mapping using energy dispersive x-ray spectroscopy (EDX) is shown in Fig. 2d. The maps confirm the uniform distribution of the elements within CCPS; additionally, an atomic ratio of 16.8: 2.2: 4.7:12.9 is detected, which is almost stoichiometric with respect to CCPS (1:1:2:6), the high-count rate of copper is due to the TEM copper grid utilized for imaging in the TEM (the full EDX spectrum is shown in Fig. S2). To mitigate the influence of copper counts in our TEM testing, we re-conducted EDX spectrum and mapping analysis on the CCPS stones used for flake exfoliation. Figure S3 illustrates the EDX spectra of a CCPS stone captured using scanning electron microscopy (SEM). The inset shows the SEM image of the analyzed area. Additionally, a table is provided, detailing both the atomic and weight percentage composition of CCPS. The detected ratio of 1:1.1:2.1:5.7 aligns well with the anticipated stoichiometric composition of CCPS (1:1:2:6).

### Electrical modulation and ionic migration in CCPS

Through systematic investigations of the ferroic compounds, CuInP_2_S_6_ and CuCrP_2_S_6_, it has been ascertained that the dominant determinant governing their electrical behavior originates from their profoundly mobile electrochemically active Cu ions within the lattice structure^25,30–33^. At ambient temperatures, these materials exhibit insulating characteristics under low applied voltage conditions. On the other hand, they demonstrate exceptionally sensitive current response to electric stimuli beyond a certain threshold voltage. This remarkable phenomenon of reversible switching between insulating and conducting states, combined with the graded local distribution of charged Cu ions, has been examined in this section. This electrical characterization bears significant advantages in facilitating the correlation between optoelectronic and ionotronic conduction processes, as further elucidated in the subsequent section dedicated to electro-optic tuning.

**Fig. 3:**
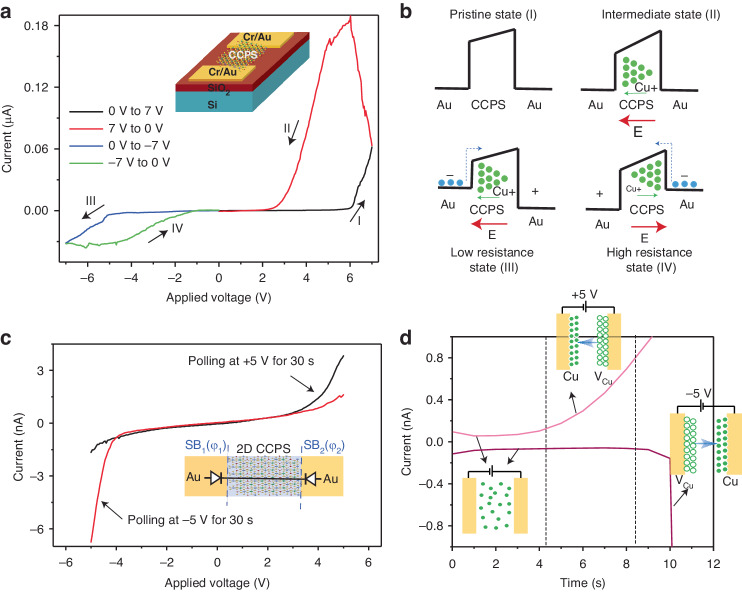
Electrical characteristics of Au/CCPS/Au structure. **a** Full I−V curves swept in the order 0 V→7 V→0 V→−7 V→0 V labeled as I, II, III, IV, inset shows a schematic of the fabricated planner Au/CCPS/Au structure, **b** Schematic of band alignment at different bias condition, **c** Forward and backward rectification states obtained by polling the voltage at ±5 V for 30 s and subsequently performed a complete cycle of the I–V characteristics, **d** Current-time (I–t) characteristics at ±5 V along with a schematic representation of Cu distribution

Figure 3a depicts the current-voltage characteristic (*I-V*) of a symmetric planar Au/CCPS/Au device, obtained with a ramping rate of 0.5 V/s. During the initial stage, a positive bias voltage is swept from 0 to 7 V, triggering a transition from a high resistive state (HRS) to a low resistive state (LRS) at a threshold voltage (V_th_) of approximately 6.0 V. However, upon reversing the positive voltage sweep (from 7 V to 0 V), the device reverts to the HRS. The presence of a hysteresis loop indicates a bidirectional threshold resistive switching (RS) effect, making the device a promising candidate for neuromorphic computing^46,47^.

Additionally, when the *I*–*V* characteristics are cycled under negative bias voltage (0 to −7 V, and then from −7 back to 0 V), similar behavior is observed compared to the forward scans under positive bias voltages. To further investigate the RS characteristics, the *I–V* response is examined with different sweep voltages ranging from ±5 to ±10 V applied to the planar Au/CCPS/Au device, as shown in Fig. S4. This result demonstrates a switchable bidirectional diode behavior accompanied by resistive switching for these voltage ranges. The switching ratio (*I*_LRS_/*I*_HRS_) reaches 0.1 µA/25 pA, equivalent to 4 × 10^3^.

The observed behavior is in accordance with prior results obtained using a conductive scanning tip, where pronounced hysteresis and rectification behavior, contingent on sweeping speed, are attributed to the migration and uneven accumulation of Cu ions^25,29^. Importantly, this process exhibits reversibility, as long as the device remains undamaged by the substantial current induced by Joule heating.

Figure 3b depicts the band diagram of the Au/CCPS/Au device under various bias voltage conditions, labeled from I to IV. In the absence of applied bias, the Cu ions are uniformly distributed throughout the CCPS phase, representing the pristine state I (the presence of a bent band structure suggests the existence of interface impurities between the gold and the semiconductor CCPS, which are likely to induce Fermi level pinning). However, upon the application of bias voltage, the Cu ions undergo migration, leading to a nonuniform distribution compared to the unbiased condition, forming the intermediate state II.

States III and IV reveal the resistive switching and bidirectional rectification phenomena. In state III, the electric field progressively increases toward the left, accelerating the migration of Cu ions in the same direction, resulting in a higher Cu accumulation at the left Au electrode. As a consequence, this electrode attains n-type characteristics, while the other electrode experiences Cu ion depletion, leading to a p-type state. Consequently, the barrier height reduces due to the increased accumulation of Cu ions at the Au/CCPS interface, which is expected to enhance carrier transport across the interface. Similarly, a corresponding trend is observed during opposite polarity sweeping, as depicted in state IV.

To further investigate the forward and backward rectification states, we polled the voltage at ±5 V for 30 s and subsequently performed a complete cycle of the *I-V* characteristics. As demonstrated in Fig. 3c, the homojunction Au/CCPS/Au exhibits dual rectification, confirming the ionic conduction and redistribution of Cu ions during opposite polarity conditions.

Moreover, Fig. 3d and Fig. S4c illustrate the time-dependent conductivity of the Au/CCPS/Au structure. It is worth noting that the initial current of 2 × 10^−12^ A exhibits a gradual increase within a few seconds to a few microamps. This observed current progression implies that the ionic conductivity of copper can be stimulated when an external electric field of sufficient strength overcomes the potential barrier. The depletion of Cu ions from the lattice results in the creation of additional vacancies, thereby elevating the possibility of ion hopping and diffusion. Consequently, the ionic conductivity experiences exponential growth over time. This trend remains consistent when the voltage polarity is reversed. As can be seen in Fig. 3d the current gets relatively larger in one direction (at −5 V) compared to the other (at +5 V) after 9 s which can be attributed to several factors (1) slight asymmetries in the interfaces with the electrodes which can lead to different behaviors under reverse or forward bias. These asymmetries might be due to variations in defect densities and interface states. Such variations can lead to different charge transport mechanisms or barrier heights at the interfaces, impacting the current levels under different polarities (2) the Schottky barrier at the Au/CCPS interface and its interaction with the applied electric field can vary depending on the polarity. This interaction can influence the drift and diffusion of copper ions, leading to differences in current magnitude (3) the behavior of traps in the CCPS, which can capture or release charge carriers, might also be influenced by the direction of the electric field. This could result in a different concentration of free carriers under different polarities, affecting the current magnitude (4) CCPS is known to be ferroelectric under applied electric field at room temperature, thus the polarization direction could influence charge carrier dynamics. The interaction of ferroelectric domains with the applied electric field might differ based on the polarity, affecting the current flow. Additionally, the voltage ramping rate in V/s affects the amount of the loops observed in the *I-V* characteristics as depicted in Fig. S4d. Notably, at a higher ramping rate of 1 V s^−1^, the Cu ions have limited time to redistribute, resulting in a smaller hysteresis loop. Conversely, when the ramping rate is reduced to 0.3 V s^−1^ there is more time for the Cu ions to migrate towards the opposite electrode. This increased migration time leads to a more pronounced variation in ion distribution, and as a result, a larger hysteresis is observed when the voltage direction is reversed. This means that the devices exhibit scanning speed dependence, indicating that ion migration is dominant. In our devices, the principal mechanism of conduction is ionic conductivity, although we also acknowledge the potential secondary role of ferroelectric domains and electronic conductivity.

### Photonic design and CCPS integration: theory and simulation

**Fig. 4:**
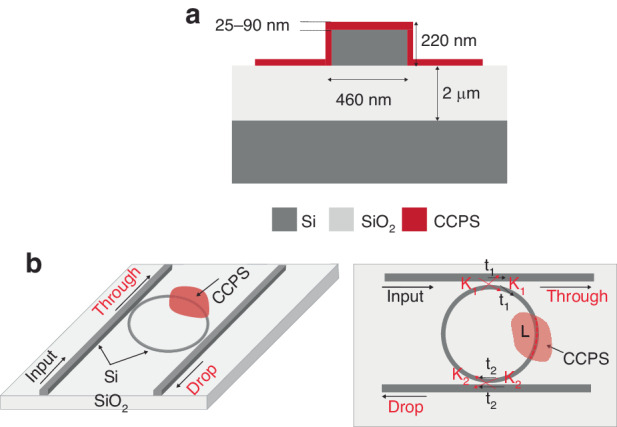
Schematic configuration of the photonic design and CCPS integration. **a** Cross-section showing design parameters, **b** 3D and cross-section of the Si-MRR design with integrated CCPS

Figure 4 depicts a schematic configuration of the hybrid integration of multilayer CCPS into SiPh circuits. In this study, SOI wafers with a 220 nm top silicon layer and a 2 µm buried oxide layer are used. The MRR cavity’s waveguide width (d) is 460 nm, and its ring radius is 45 µm. A 100 nm gap spacing is used to couple light to the micro-resonator from a bus waveguide and collected at the through port output.

Incorporating a CCPS material on top of the ring waveguide offers the ability to control light propagation in the “add” and “through” ports of the resonator by adjusting the real part of the CCPS’s refractive index via electro-optic tuning. Light passing through the waveguide is coupled to the top CCPS layer evanescently, where the coupling strength is influenced by the thickness of the CCPS. The expressions for the transmission of the resonator’s exit ports can be formulated as follows^48^:

1 $${T}_{{through}}=\frac{{t}^{2}{\alpha }^{2}-2{t}_{1}{t}_{2}\alpha \cos \theta +{t}_{1}^{2}}{1-2{t}_{1}{t}_{2}\alpha \cos \theta +{({t}_{1}{t}_{2}\alpha )}^{2}}$$

2 $${T}_{{drop}}=\frac{\left(1-{t}_{1}^{2}\right)(1-{t}_{2}^{2})\alpha }{1-2{t}_{1}{t}_{2}\alpha \cos \theta +{({t}_{1}{t}_{2}\alpha )}^{2}}$$

Where α is the attenuation factor, *θ* is the phase factor, *t*_1_ and *t*_2_ are coupling parameters, *α*, and *θ* can be expressed as:

3 $$\alpha =\exp \left(-\frac{2\pi }{\lambda }\left[{k}_{{eff},{wg}}\left(2\pi R-{L}_{{CCPS}}\right)+{k}_{{eff},{CCPS}}{L}_{{CCPS}}\right]\right)\approx \exp \left(-\frac{2\pi }{\lambda }{k}_{{eff},{CCPS}}{L}_{{CCPS}}\right)$$

4 $$\theta =\frac{2\pi }{\lambda }\left[{n}_{{eff},{wg}}\left(2\pi R-{L}_{{CCPS}}\right)+{n}_{{eff},{CCPS}}{L}_{{CCPS}}\right]$$

The effective imaginary and real components of the refractive index of the Si waveguide with (without) CCPS are denoted by *k*_*eff*_,_*CCPS*_ (*k*_*eff*_,_*wg*_) and *n*_*eff*_,_*CCPS*_ (*n*_*eff*_,_*wg*_), respectively. *R* is the radius of the ring waveguide, while *L*_CCPS_ is the length of the integrated CCPS flake.

**Fig. 5:**
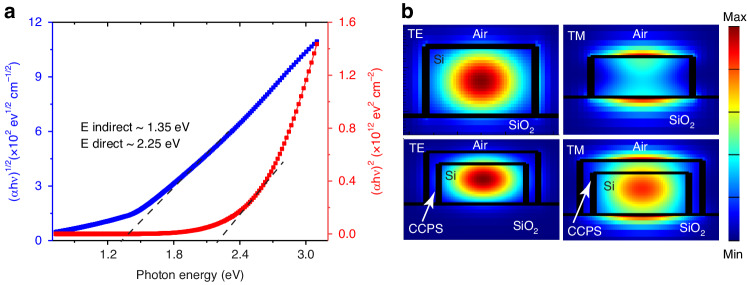
Optical properties and guided light/CCPS interaction. **a** Direct and indirect transition Tauc plots of the absorption coefficient derived from high-resolution spectroscopic Ellipsometry, **b** Electric field profiles (|E|^2^) of TE and TM modes of bare Si waveguide (top panel) and 65  nm CCPS on Si (bottom panel) at 1310 nm

The optical parameters, namely, refractive index (*n*) and extinction coefficient (*k*) of exfoliated CCPS on 300 nm SiO_2_/Si substrate and on Si-MRR are extracted from high-resolution spectroscopic ellipsometry measurements (more details on ellipsometer measurements, and n, *k* values for integrated CCPS on Si-MRR and flat surface are included in the supplementary material, Figs. S5, S6). The extinction coefficient is linked to the absorption coefficient (*α*) as *α* = 4π*k*/λ, hence, the absorption coefficient can be calculated and fitted to Tauc’s equations, as shown in Fig. 5a. The extracted indirect bandgap is about 1.35 eV, whereas the direct bandgap is higher by 0.9 eV. It is worth mentioning that no photoluminescent emission was observed at room temperature. Furthermore, these values are consistent with the literature and the low optical loss (transparency) in the NIR range (1280–1360 nm), as will be discussed in the next section^25^.

The interaction between guided light and the multilayer CCPS plays a pivotal role in designing optimal optoelectronic devices on the SiPh platform^49^. Consequently, numerical and experimental investigations were conducted to test the variations in the effective refractive index (*n*_eff_) and optical losses of the hybrid Si/CCPS structures. These calculations were executed for different CCPS thicknesses, with a specific focus on the NIR wavelength from 1280 nm to 1360 nm. The range of CCPS thickness (30 nm to 90 nm) used in the simulation is aligned with our experimental tests. The measured values of refractive index (*n*) and extinction coefficient (*k*) of multilayer CCPS were then fed into the mode analysis of Lumerical Mode Solver Software.

In Fig. 5b, the electric field profiles (|E|^2^) of the fundamental guided modes are displayed for both a bare silicon waveguide and integrated structures with a 65 nm CCPS layer at 1310 nm. As observable, the silicon waveguide supports quasi-TE (transverse electric) and quasi-TM (transverse magnetic) modes.

Furthermore, the mode evanescent field effectively overlaps with the multilayer CCPS flake for both polarizations, where the fraction of the optical mode power in CCPS is ~11% and ~12% for TE and TM polarization, respectively. The mode profile and overlap factor for other CCPS thicknesses are included in Fig. S7.

### Device fabrication and testing

**Fig. 6:**
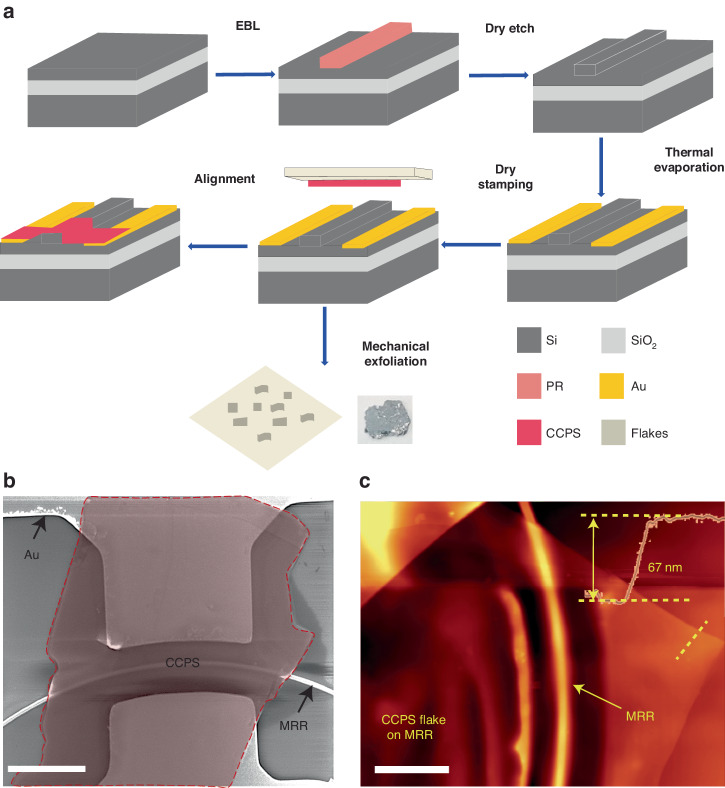
CCPS integration on SiPh platform. **a** Schematic representation of the fabrication and CCPS integration process flow, **b** SEM image of the transferred CCPS on MRR, **c** AFM image scan, the dashed yellow line shows a thickness of ~67  nm CCPS. Bars in (**b**, **c**) are 10  µm and 6 µm, respectively

The multilayer CCPS flakes were obtained through mechanical exfoliation from their bulk crystals, which were commercially available from HQ Graphene. In this investigation, we systematically transferred CCPS flakes with varying thicknesses ranging from 30 nm to 90 nm onto the MRR structure utilizing a deterministic dry transfer process^50–52^. Figure 6a shows a schematic representation of the fabrication and CCPS integration process. The hybrid Si/CCPS MRR’s morphology is depicted in Fig. 6b through SEM images. Evidently, the CCPS flakes exhibit good alignment and conform to the photonic structure beneath, facilitating strong light/CCPS coupling. To quantitatively assess the thickness of the transferred flakes, atomic force microscopy (AFM) was employed, and the results are depicted in Fig. 6c. Additionally, Fig. S8. displays a 3D reconstructed AFM scan, showcasing the conformal coverage and strong adhesion of the CCPS flakes on both the top surface and sidewalls of the MRR waveguide. Further details on AFM scan, cross-section and 3D reconstructed images with strain effect on our device are included in the supporting information Figs. S9, S10.

### Optical transmission measurements

**Fig. 7:**
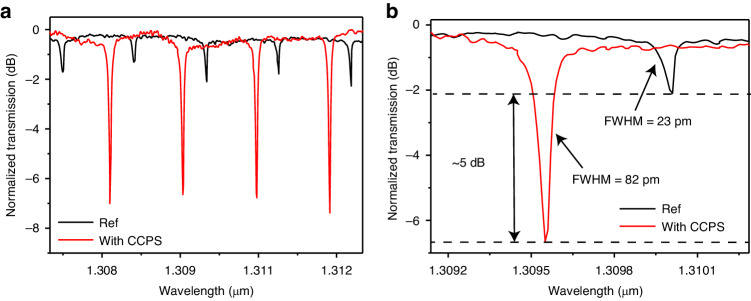
Passive optical measurements. **a** Measured transmission spectra of TE mode in Si/CCPS hybrid MRR with a radius of 45 μm and CCPS thickness of ~67 nm a long with bare Si-MRR as a reference, **b** Zoom-in image of the MRR resonances with and without CCPS showing the change in FWHM of the resonance dips, dashed line is the Lorentz fitting while solid line is the experimental transmission spectra

To experimentally verify the modulation of the hybrid integrated waveguide’s refractive index and the optical losses, the optical transmission spectra of the CCPS-integrated MRR were measured. For this study, we used a tunable laser operating at SWIR wavelengths to edge-couple TE-polarized light into the MRR through a lensed fiber. The output response was collected by another lensed fiber and detected using a power meter. The light in the Si waveguide is evanescently coupled into the CCPS flake. Figure 7a displays the resonance dips of the MRR before (described as reference) and after integrating a CCPS flake with a thickness of ~65–67 nm and a coverage length of ~28 μm. The corresponding response for the TM mode can be found in the supplementary information Fig. S11a. Additionally, we examined the MRR’s response at various CCPS coverages and included their transmission spectra in the supplementary information Fig. S11b. Intriguingly, the incorporation of CCPS into the MRR structure results in strong light-matter interaction with ~ 5 dB higher dips extinction ratio (ER) compared to bare silicon and low optical losses (see Fig. 7a, b).

Moreover, we experimentally extracted the optical losses due to the multilayer CCPS by analyzing the variation in the MRR’s Q-factor^53^. As illustrated in Fig. 7b, the loaded quality factor (λ_0_/FWHM) decreased from ~5 × 10^4^ (for the bare Si-MRR, the value averaged over 5 tested rings) to ~2 × 10^4^ (for the hybrid CCPS/Si-MRR with a flake thickness of 67 nm and interaction length of ~28 µm, the value averaged over 5 CCPS loaded rings). This observation highlights the low-loss characteristics of CCPS in the SWIR wavelength. To evaluate the additional optical losses induced by the integrated CCPS flakes in the TE polarization, we calculated the difference between the loss of the reference ring and that obtained in the hybrid structure (CCPS/Si). These losses primarily originate from reflections and scattering at the coupling interface between the passive waveguide (air/Si) and the hybrid region, involving mode mismatching losses and flake irregularities. Negligible losses are attributed to material absorption in the SWIR.

The extracted optical losses amount to 2.7 dB cm^−1^. Further details on the calculation are available in the supplement note 1. This value is comparable to those of lithium niobite and some barium titanate devices^39,40,54^, in addition to monolayer TMDs optimized to operate on their transparency window (O and C optical bands) for different near-infrared electro-optic applications^7^ (see Table 1).

**Table 1:** Summary of electro-optic phase shifter fabricated based on ferroelectric materials (BaTiO_3_, LiNbO_3_) and electrostatic doping of TMDs

Material platform	Photonic structure	Length of modulation area (µm)	Tuning efficiency (pm V^−1^)	V_π_ L (V cm)	Optical propagation loss (dB cm^−1^)	Ref.
LiNbO_3_ (LN)	Si/LN Mach–Zehnder modulators (MZMs)	5000	-	2.2	0.98	^56^
BaTiO_3_ BTO)	Hybrid BTO-SiO_2_ racetrack resonator on SiPh	100	923	0.45	10.5	^57^
BaTiO_3_ (BTO)	BTO-SiN racetrack resonator	150	-	-	9.4	^38^
TMD	Monolayer WS_2_ on SiN-MZI	500	-	0.8	1.5	^7^
TMD	Few-layer MoS_2_ on SiN microring resonator	-	29.42	0.69	-	^16^
Ferroionic 2D materials	Multilayer ~67 nm CCPS on microring resonator	20	8.3	0.25	2.7	This work

### Electro-optic tuning

**Fig. 8:**
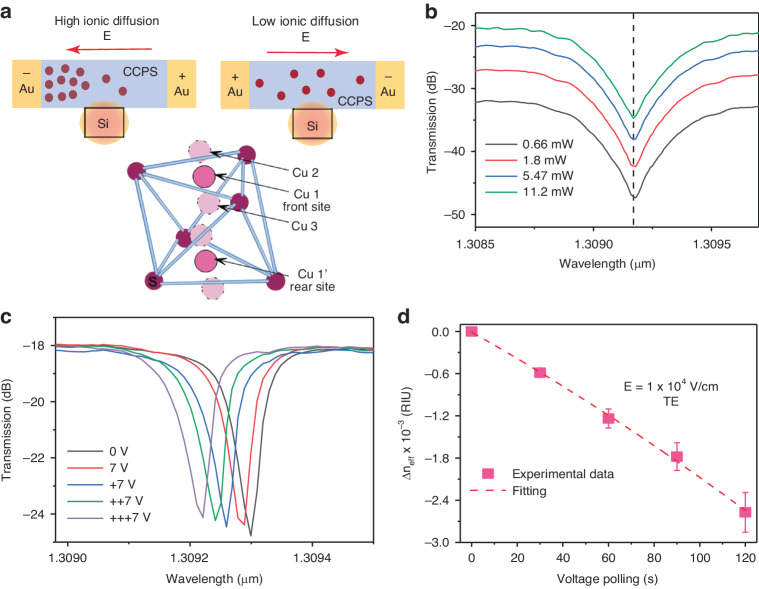
Electro-optical testing of hybrid CCPS/Si-MRR. **a** Visualized illustration of copper redistribution in CCPS/Si heterostructure upon application of electric field along with the movements of Cu in the sulfur cage. **b** The transmission spectra of MRR at different input optical power. **c** The transmission spectra of MRR at a constant voltage of 7 V as the polling time progressed. **d** The change in effective index of refraction versus the polling time at *E* = 1 × 10^4^ V cm^−1^

To probe the ionic migration-induced electro-optical response of CCPS, a hybrid Si–CCPS MRR platform was utilized. A 20–35 μm arc length of CCPS was integrated onto the ring. As depicted in Fig. 6b, the flakes are stamped into pre-patterned 100 nm Au/10 nm Cr electrodes separated by 3.25 μm on both sides of the waveguide (more details are included in supplement note comparative analysis and device testing). It is worth mentioning that in passive measurement the coverage length is all the length covered by the flake (i.e. can be >20 µm), however, in active tuning the max active length is 20 µm (defined by the electrode length). This cavity is critically coupled into a Si bus waveguide, making the ring’s transmission spectrum highly sensitive to minute phase and absorption variation within the cavity. We tune the effective index of the optical mode by harnessing the migration of Cu ions driven by the electric field within the CCPS, as illustrated in Fig. S12 of our experimental setup. Figure 8a illustrates the impact of an applied potential difference across the electrodes connected to the CCPS, resulting in a progressive redistribution of Cu ions. This phenomenon consequently instigated a discernible alteration in both the copper ions concentration and the CCPS electrical conductivity. Due to the soft nature of Cu–S bond, it allows the Cu ion hop between the intralayer and interlayer sites and even across the vdW gap interlayer under the influence of an electric field (see Fig. 1). Hence, optical tuning can be achieved by utilizing the reversible process of Cu ions extraction from sulfur octahedra through van der Waals gaps and re-intercalation into layers, a process reliably controlled by the applied voltage.

The effect of the applied bias on resonance wavelength shift was carried out at SWIR. To mitigate any potential influence from the laser input power, the optical power delivered to the Si/CCPS chip was systematically varied from 0.66 mW (−1.8 dB) to 11.2 mW (10.5 dB), while maintaining the device bias at 0 V. As evident in Fig. 8b, there is no discernible change in the positions of the resonance peaks for both polarizations. Accordingly, the propagating light induces no noticeable thermal dissipation in the device. Thus, we limited the laser power in all subsequent measurements to less than 11.2 mW (10.5 dB). Note that the 10.5 dB is the laser input power which is not the power delivered to the CCPS/Si-MRR. The power delivered to CCPS/Si-MRR is −1.5 dB [10.5 dB (laser input power)−12 dB (insertion loss per facet)].

Figure 8c depicts the transmission spectra for TE polarization at bias voltages of 0 V and 7 V. The spectra were recorded with a constant voltage of 7 V as the polling time progressed. The polling instances are labeled as +7, ++7, +++7, spaced 30 s apart from each scan. Across all scans, a consistent blue shift in the resonance wavelengths was observed for all fabricated devices (see Fig. S13 and supplement note for the underlying mechanism of index of refraction change). This blue shift implies an electro-optical modulation of the refractive index of the combined Si/CCPS guiding structure, given that thermal heating typically results in a red shift in the Si-MRR resonance wavelength^38,55^ Remarkably, the application of a bias across the device does not affect the extinction ratios and the resonance linewidth. Therefore, the active migration of Cu ions has no impact on the imaginary part of the refractive index.

Figure 8d depicts the change in the effective index of refraction as a function of polling time at 7 V (*E* = V/d). Figure S14 shows the resonance shift as a function of increasing voltage, from this, we estimate a tuning efficiency (δλ/δV) of −8.3 pm V^−1^. The active migration of Cu ions demonstrates the ability to electrically tune the effective refractive index on the order of 2.8 × 10^−3^ RIU (see supplement note 2). Moreover, the half-wave voltage-length product is *V*_π_*L* = 0.5·L·FSR/(δλ/δV) ≈ 0.25 ± 0.07 V cm (FSR = 1.4 nm, *L* = 20 µm) which is lower than the phase shifter demonstrated based on TMDs^7,16,17^ (see Table 1). It’s important to highlight that our experimental observations with various CCPS devices revealed quicker switching post pre-poling. Pre-poling in CCPS aligns the ions advantageously, creating defined electrical paths, thereby shortening the time for ions to reach the desired state under an applied electric field. This phenomenon mirrors what is seen in materials like ferroelectric PZT (Lead Zirconate Titanate), extensively used in electro-optic applications.

**Fig. 9:**
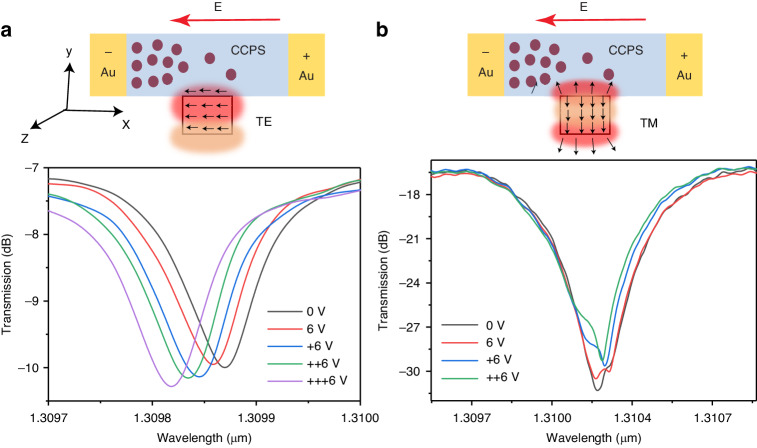
Electro-optic response of the hybrid MRR structure. **a** TE mode; **b** TM mode at a constant voltage of 6 V as the polling time progressed, along with graphic representation showing the direction of electric field components for both polarizations

Figure 9a, b show the transmission spectra for the TE and TM modes, respectively, under varying polling times at an applied voltage of 6 V. This voltage induces the migration of Cu ions in the integrated CCPS/Si-MRR. In the case of the TE mode (Fig. 9a), a pronounced wavelength shift is observable, while the TM resonance mode (Fig. 9b) displays no discernible shift. This observation suggests that the influence of ionic conductivity and resistance change is significant only when the optical electric field aligns with the CCPS. Specifically, this effect is pronounced when the E_x_ component in TE polarization aligns with the applied electric field, as illustrated in the schematic representation in Fig. 9a, b. we further expanded our testing to include the 1500–1600 nm wavelength range. Figure S15a presents the resonance dips of the MRR both before and after the integration of a CCPS flake. This flake, ~54 nm thick and covering ~31 μm, was tested in the 1500–1600 nm range. Similar to the results observed in the 1280–1360 nm range, the integration of CCPS into the MRR resulted in a pronounced light-matter interaction, characterized by higher extinction ratio (ER) dips compared to bare silicon and minimal optical losses.

Additionally, Fig. S15b displays the transmission spectra for TE polarization under bias voltages of 0 V and 6 V, recorded continuously at 6 V as the polling time increased. Throughout these scans, a consistent blue shift in resonance wavelengths was noted, indicating an electro-optical modulation of the refractive index within the Si/CCPS guiding structure at these wavelengths. These findings suggest that our devices exhibit response across SWIR wavelengths.

## Discussion

we demonstrated a novel avenue for active light manipulation through the utilization of ferroionic 2D CCPS material. When integrated into SiPh microring resonators, these materials exhibit a remarkable ability to finely tune the effective index of refraction without any amplitude chirp. This is attributed to the adjustable electrical conduction that originates from the reversible accumulation or removal of mobile Cu ions at the metal-semiconductor interface. Results show that electrically driven Cu ions can tune the effective refractive index on the order of 2.8 × 10^−3^ RIU (refractive index unit) while preserving extinction ratios and resonance linewidth. Additionally, the devices feature remarkably low optical losses and excellent half-wave voltage-length product of *V*_π_*L* = 0.25 V cm, which is lower than the phase shifter demonstrated based on TMDs. We further determine the electro-optic tuning sensitivity to light polarization. It is evident that the influence of ionic conductivity and resistance change is significant only when the optical electric field aligns with the Cu ions migration direction (same as the applied field). Strong light-matter interaction of CCPS offer a new pathway to address challenges in achieving pure phase modulation while enabling efficient and versatile light manipulation. This hold potential for applications, including optical switching, environmental sensing and metrology, optical imaging systems, and neuromorphic systems in light-sensitive artificial.

## Materials and methods

### Materials supply

CuCrP_2_S_6_ bulk crystals were purchased from a hq graphene vendor (https://www.hqgraphene.com/).

### Scanning electron microscopy

Photonic chips were mounted on an SEM stub using carbon tape and imaged under high vacuum mode by using a (FEI) Quanta 450 field emission scanning electron microscope with an electron energy of 10 KV.

### Atomic force microscopy

It was performed using a WITec AFM module integrated with a research-grade optical microscope in the tapping mode. The cantilever tip (Scanasyst-air) had a radius of 7 nm, a force constant of 0.2 N m^−1^, and a resonance frequency of 14 kHz.

### Raman spectroscopy

Confocal micro-Raman (WItec alpha 300) with a laser excitation source of 532 nm was precisely subjected to the flake using a 100× objective lens with a spot size of ~0.7 µm. To avoid any damage to the sample, a low laser power was applied.

### Transmission electron microscopy

A high-resolution analytical scanning/ transmission electron microscope (FEI Talos F200X) is operated at 200 keV. It combines high-resolution scanning/transmission electron microscope and TEM imaging with a four-quadrant energy dispersive X-ray spectrometer (EDS) for elemental and compositional mapping. CCPS flakes were exfoliated using scotch tape and transferred into PDMS. After that, the flakes are stamped into the TEM copper grid. Prior to imaging, the copper grid was placed in plasma cleaning to remove any contamination left from the stamping process.

### Mode analysis and FDTD simulation

The electric field profile in the silicon waveguide and the beam propagation were calculated using the MODE Solutions eigenmode solver, a simulator within Lumerical’s Device Multiphysics Simulation Suite.

### Spectroscopic imaging ellipsometer

The optical parameters of multilayer CuCrPS were determined by Accurion’s Imaging Ellipsometry (https://accurion.com/company). This system combines optical microscopy and ellipsometry for spatially resolved layer-thickness and refractive index measurements. It is highly sensitive to single- and multilayer ultrathin films, ranging from mono-atomic or monomolecular layers (sub-nm regime) up to thicknesses of several microns. Additionally, Imaging Ellipsometers perform layer-thickness measurements with a spatial resolution down to 1 µm. The ellipsometric parameters (Psi (*ψ*) and Delta (*Δ*)) were fitted using EP4 model software.

### Stamping of CCPS on the photonic chip

Multilayered flakes are exfoliated using scotch tape and transferred onto the PDMS substrate. By using the controllable dry transfer method, the selected CCPS flakes are transferred onto the Si waveguides. More details on the deterministic transfer process are included in the supplementary material.

### Electrical measurements

A curve tracer/power device analyzer / (Agilent B1505A) was used to control the biases and measure the I-V characteristics via a pair of standard DC electrical probes under dark conditions. All electrical measurements were performed in air at room temperature.

### Optical characterization

The optical transmission was tested by edge coupling the light into the device structure through lensed fiber using a tunable laser operating at the SWIR band (Keysight 8164B Lightwave Measurement System). The output response from the devices is collected by an output lensed fiber and detected by a power meter. The light polarization (TE/TM) was calibrated using reference rings fabricated on the same chips with identical geometries. The output optical power intensities were calibrated before the device testing using a standard photodiode power sensor. All optical measurements were performed in air at room temperature.

### Supplementary information

Supplemental Material
