# Supplementary material for: Electro-optic tuning in composite silicon photonics based on ferroionic 2D materials
Source: Light Sci Appl. 2024 Apr 19;13:92. doi: 10.1038/s41377-024-01432-2 (PMC11031603; doi:10.1038/s41377-024-01432-2)
Supplement: Supplementary file 1 — Supplemental Material [file 41377_2024_1432_MOESM1_ESM.docx]

**Supplementary Information for**

**Electro-Optic Tuning in Composite Silicon Photonics Based on Ferroionic 2D Materials**

Ghada Dushaq *, Solomon Serunjogi, Srinivasa R. Tamalampudi, and Mahmoud Rasras*

Department of Electrical and Computer Engineering, New York University Abu Dhabi, P.O. Box 129188, Abu Dhabi, United Arab Emirates

[ghd1@nyu.edu](mailto:ghd1@nyu.edu), [mr5098@nyu.edu](mailto:mr5098@nyu.edu)

**This file includes:**

**Figure S1.** Structural characteristics: HRTEM of multilayer CCPS

**Figure S2.** Elemental analysis: EDX spectra of multilayer CCPS flakes using TEM

**Figure S3.** Elemental analysis: EDX maps and spectra on CCPS stones using SEM

**Figure S4.** Electrical characteristics of Au/CCPS/Au structure

**Figure S5.** Optical parameters measurements

**Figure S6.** Optical images during wavelength scan in ellipsometer measurements

**Figure S7.** Mode simulation and confinement factor

**Figure S8.** CCPS integration

**Figure S9.** CCPS integration and structural topology

**Figure S10.** CCPS integration and strain effect

**Figure S11.** Optical transmission measurements

Supplement note 1 **Optical loss in CCPS using microring resonator**

Supplement note 2 **Change in the effective index of refraction calculation**

**Figure S12.** Experimental setup of electro-optic tuning of CCPS/Si MRR

Supplement note 3 **Underlying mechanism of refractive index change**

**Figure S13.** Electro-optic response of composite MRR

**Figure S14.** Resonance shift as a function of applied voltage

**Figure S15.** Electro optic tuning at 1500 nm-1600 nm

Supplement note 4 **Comparative analysis and device testing**

**Figure S16.** Optical microscopy image of the photonic chip as fabricated

**Figure S17.** Scanning electron microscopy images of transferred and tested devices during our experiments

**References**

**
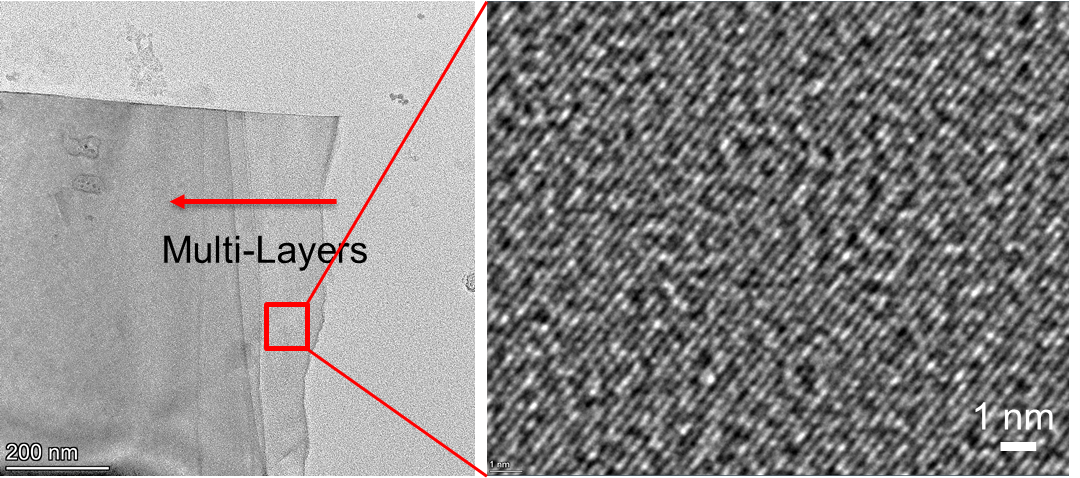
**

**Figure S1.** High-resolution transmission electron microscopy (HRTEM) of multilayer CCPS captured at the red squared area.

**
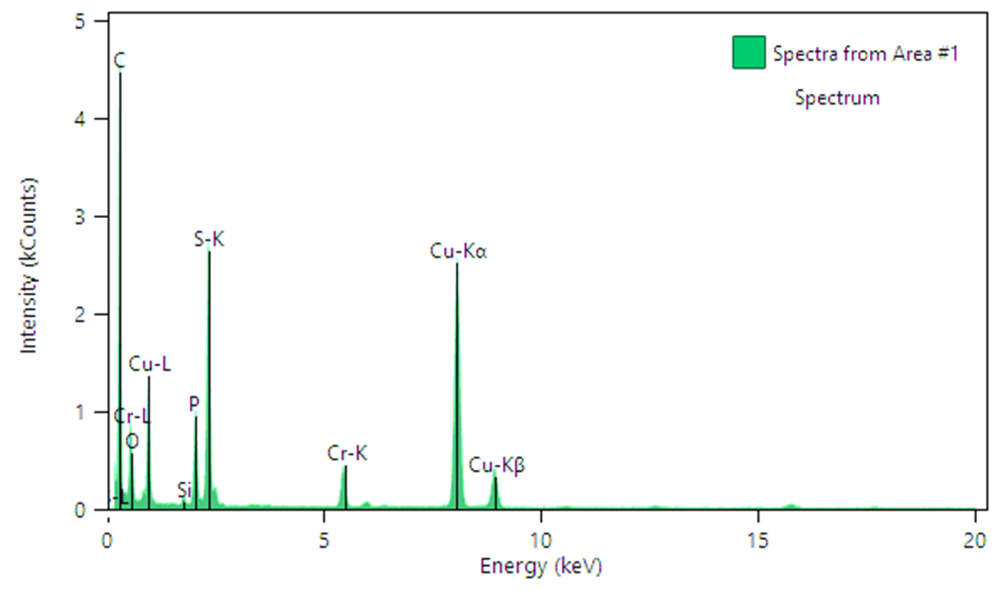
**

**Figure S2.** Energy dispersive X-ray (EDX) spectra of multilayer CCPS using TEM.

**
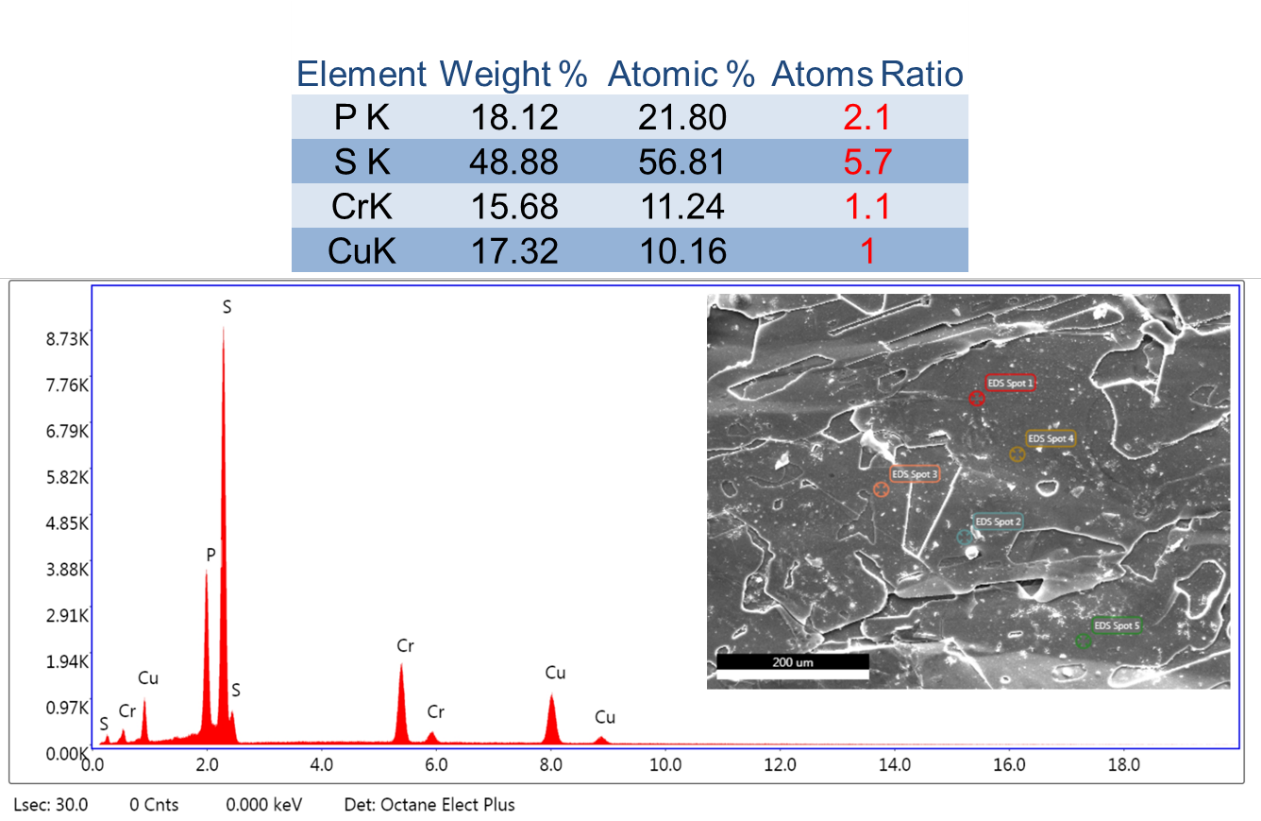
**

**Figure S3.** Energy Dispersive X-ray (EDX) analysis performed on a CCPS stone using Scanning Electron Microscopy (SEM), with the inset displaying the SEM image of the area analyzed. Accompanying this is a table that presents the atomic and weight percentage composition of the CuCrPS.

**Electrical characteristics of Au/CCPS/Au structure**

**
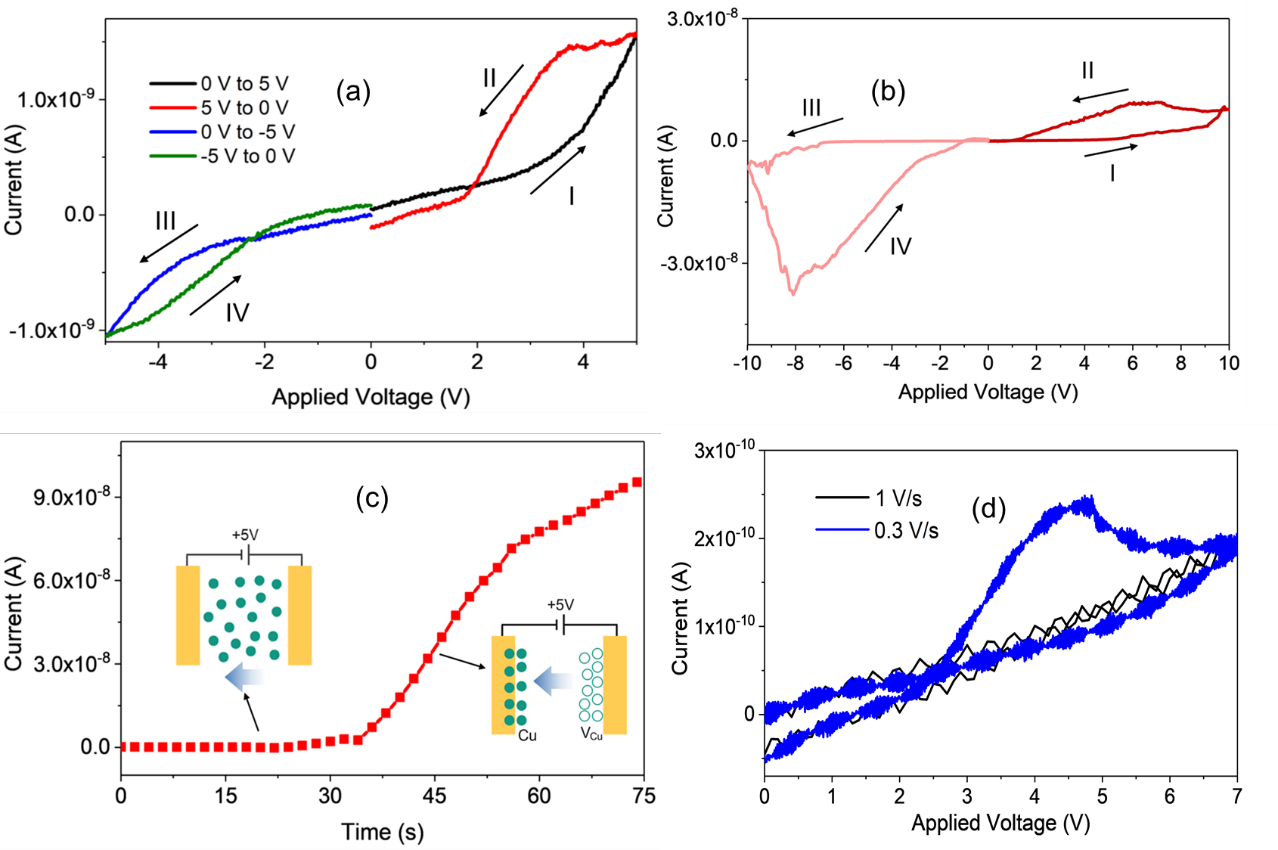
**

**Figure S4.** Electrical characteristics of Au/CCPS/Au structure (a)-(b) full I−V curves (a) swept in the order 0 V→ 5 V → 0 V → -5 V → 0 V (b) swept in the order 0 V→ 10 V → 0 V → -10V → 0 V labeled as I, II, III, IV (c) current-time (I-t) characteristics at +5 V along with schematic representation of Cu distribution (d) positive I-V cycle characteristics at different voltage scan rate.

We found that devices with enhanced ion migration exhibited larger currents and wider switching windows at slower scanning speeds (Fig.S4d), but this effect was less pronounced under fast scanning. Our results with two scanning rates (slow: 0.3 V s^-1^; fast: 1 V s^-1^) are consistent with observations in Nat. Commun. 13, 574 (2022)^1^. As expected, devices exhibiting ion migration showed a scan rate dependence. Notably, at a higher ramping rate of 1 V s^-1^, the Cu ions had limited time to redistribute, resulting in a smaller hysteresis loop. Conversely, at a reduced ramping rate of 0.3 V s^-1^, there was more time for the Cu ions to migrate towards the opposite electrode, leading to a more pronounced variation in ion distribution and, consequently, a larger hysteresis when reversing the voltage direction. This discrepancy suggests an effective approach to distinguish whether ion migration is the sole contributing factor in the device. The devices reported in our manuscript exhibit scanning speed dependence, indicating that ion migration is dominant.

**High-resolution imaging ellipsometry measurements**

**
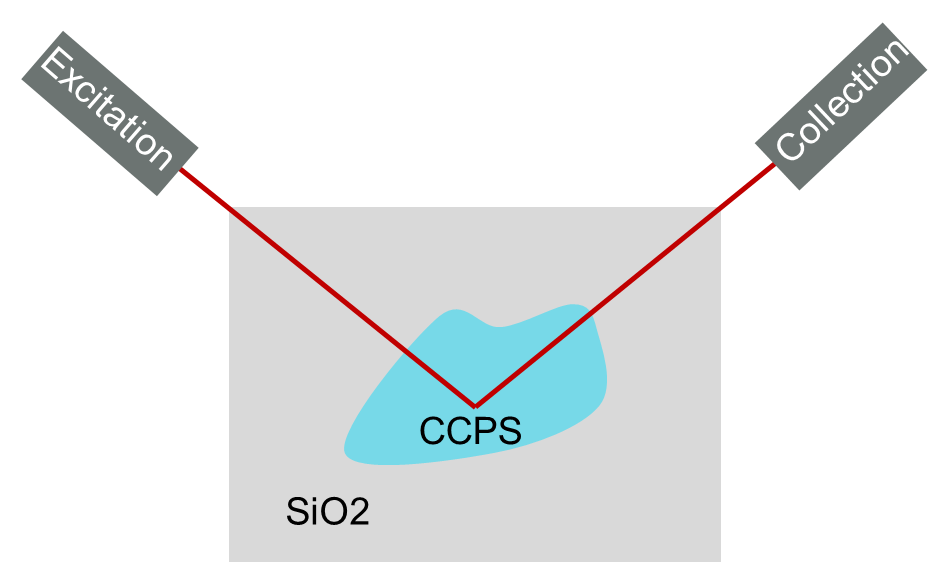
**

We use spectroscopic ellipsometry on exfoliated crystals of known thickness (measured by AFM) to estimate the optical properties (the refractive index and the extinction coefficient) of CCPS^2–5^. We measure the complex reflectance ratio for CCPS's amplitude (Ψ) and phase difference (∆), which are presented in Figure S4 below. In order to determine the complex refractive index, the experimental data points (black color) are fitted with several analytical models (red color). The bare SiO_2_/Si substrate located directly next to each investigated layered material was used to collect ellipsometric data in order to reduce substrate-induced uncertainty in the estimation of the complex refractive indices. By using known Si and SiO_2_ refractive indices to match this data, a measurement of the thickness of the SiO_2_ layer was obtained. We combine a Lorentzian oscillator-based model with a Tauc-Lorentz oscillator model to explain the exciton resonances. Table S1 provides a description of the fitting parameter.

**
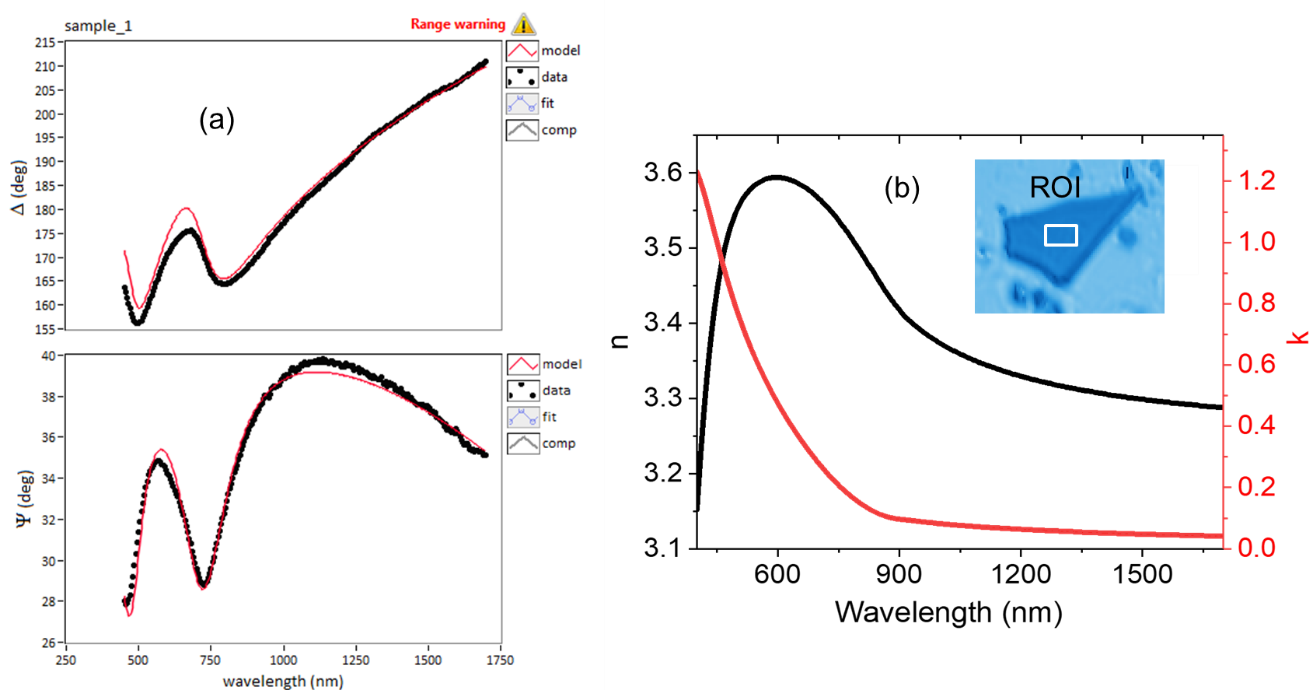
**

**Figure S5.** Optical parameters extracted from EP4 software (a) ellipsometric spectra of Delta-Psi (b) optical parameters n, k values, inset shows CCPS flake region of interest (ROI).

**Table S1:** Fitting parameters

|  | Best Fit | +/- | Units |
| --- | --- | --- | --- |
| Thickness | 62.2 | 0.0 | nm |
| Eps1 | 5.441 | 0.056 |  |
| Eps2 | 0.000 | 1.000 |  |
| Frequency (Lorentz #1) | 3.214 | 0.013 | eV |
| Strength (Lorentz #1) | 53.441 | 1.546 | eV^2^ |
| Damping (Lorentz #1) | 1.641 | 0.028 | eV |
| Frequency (Lorentz #2) | 7.936 | 5.995 | eV |
| Strength (Lorentz #2) | 10.000 | 1.000 | eV^2^ |
| Damping (Lorentz #2) | 3.000 | 1.000 | eV |
| A (Tauc-Lorentz) | 5.000 | 1.000 | eV |
| E0 (Tauc-Lorentz) | 5.000 | 1.000 | eV |
| Gamma | 1.857 | 2.110 | eV |
| Eg | 2.143 | 0.823 | eV |
| RMSE | 1.472 |  |  |

we gathered data for both exfoliated flakes on a plane substrate (300 nm SiO_2_/Si) and those integrated on Si-MRR, as illustrated by the optical images captured during the wavelength scan in Fig.S6. Given that the resolution of our Accurion’s imaging ellipsometry is approximately 1 µm, we were unable to discern any variance in the experimental optical parameters between the flake positioned on the ring (with a waveguide width of 460 nm) and that on a flat surface. Considering that optical testing is limited by the diffraction limit of light, and our waveguide width falls below our measurement resolution, we surmise that the region of interest (marked by the green square in Fig.S6b) provides an average value for that specific area.


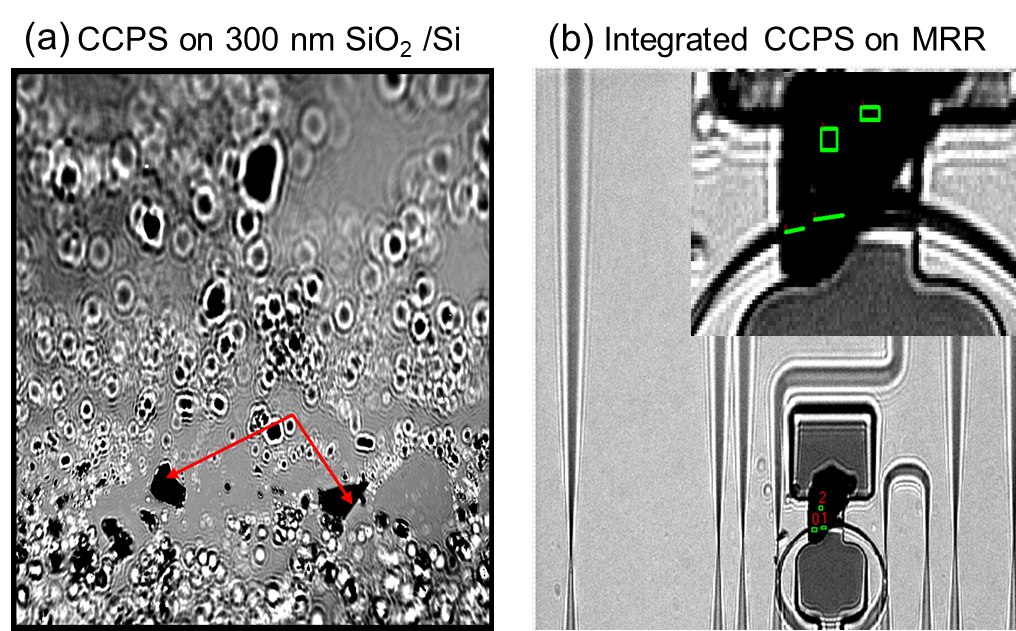


**Figure S6.** Optical images captured during the wavelength scan of the complex refraction coefficient using high-resolution Accurion’s imaging ellipsometry (a) CCPS on 300 nm SiO_2_/Si substrate (b) Integrated CCPS on Si-MRR.

**Mode and Beam propagation simulation**

**
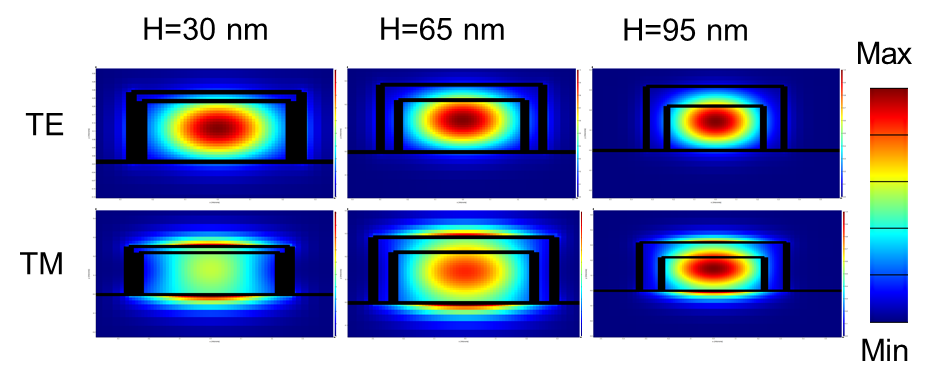
**

**Figure S7.** Electric-field profiles (|E|^2^) of TE and TM modes of CCPS with thicknesses of 30 nm, 65 nm, and 95 nm on Si at 1310 nm.

**Table S2.** Optical confinement factor of TE and TM for different CCPS thickness at 1310 nm.


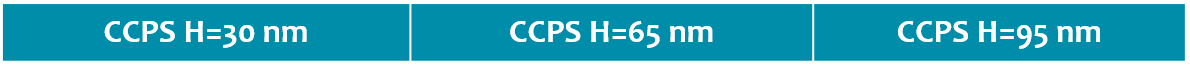


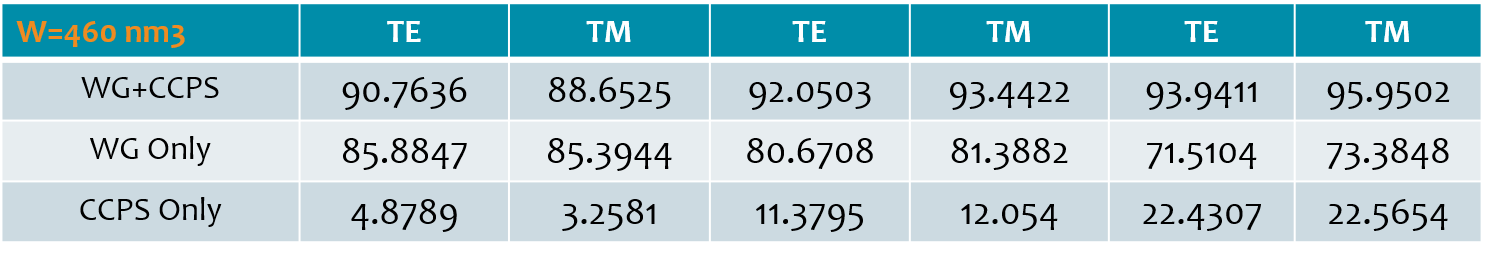


**Bare Waveguide**


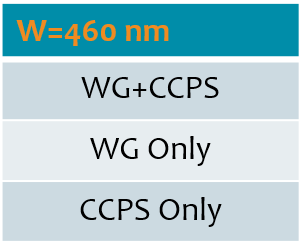

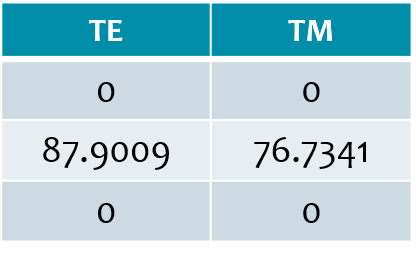


**CCPS integration into SiPh**

**
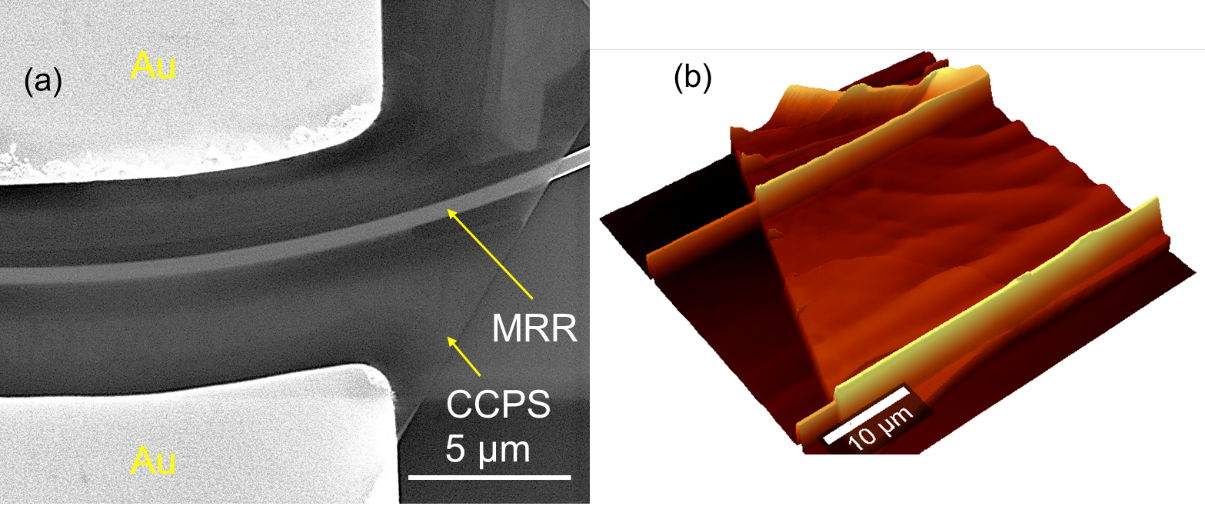
**

**Figure S8.** CCPS integration (a) SEM image of the transferred CCPS on MRR (b) 3D reconstructed AFM image scan.


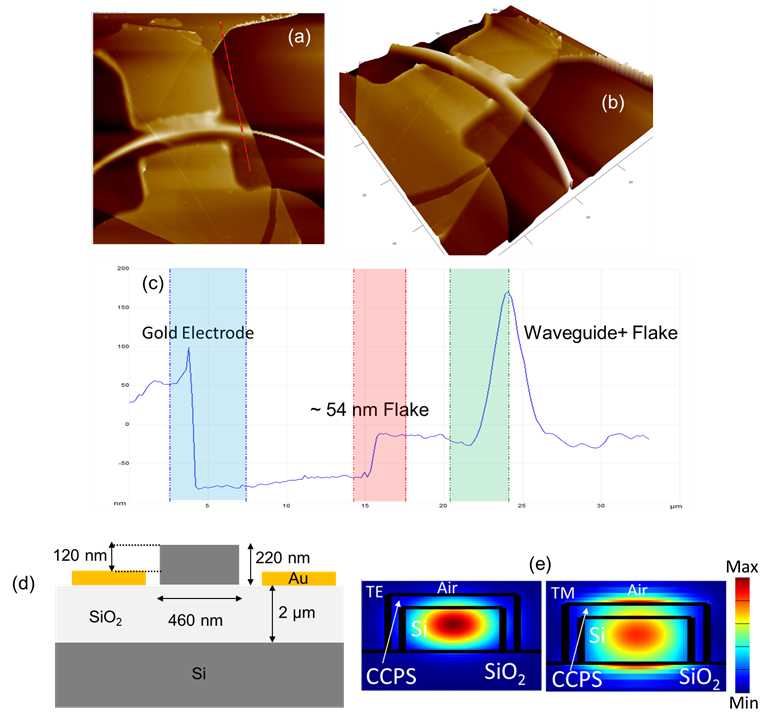


**Figure S9**. (a) Atomic Force Microscopy (AFM) scan of a 67 nm CCPS layer integrated on a Microring Resonator (MRR) (b) 3D reconstructed image derived from the AFM scan in (a), depicting the topographical detail of the CCPS on the MRR (c) Cross-sectional view along the dashed red line in (a), illustrating the height variations across the device structure (d) Schematic representation showing the height differential between various components before the integration of the 67 nm CCPS layer (e) Electric-field intensity profiles (|E|^2^) for both Transverse Electric (TE) and Transverse Magnetic (TM) modes of a 65 nm CCPS layer on a Silicon substrate, captured at a wavelength of 1310 nm.

We believe that a gap of about 50 to 70 nm might exist at the side walls. However, simulations and experimental optical transmission results indicate that the evanescent field of the TE mode predominantly interacts with the top face of the CCPS. This interaction is significant due to the slightly similar refractive index of CCPS (n ∼ 3.35) compared to silicon (n ∼ 3.4) at 1310 nm, resulting in minimal field presence at the sidewalls. The electric-field profiles (|E|^2^) of the TE mode for the Si waveguide loaded with CCPS demonstrate this interaction, leading to a substantial increase in the extinction ratio (ER) – around 5 dB higher compared to bare silicon, as shown in Figs. 7a and 7b of the paper. This strong light-matter interaction underscores the efficiency of our design, even considering the gap at the sidewalls


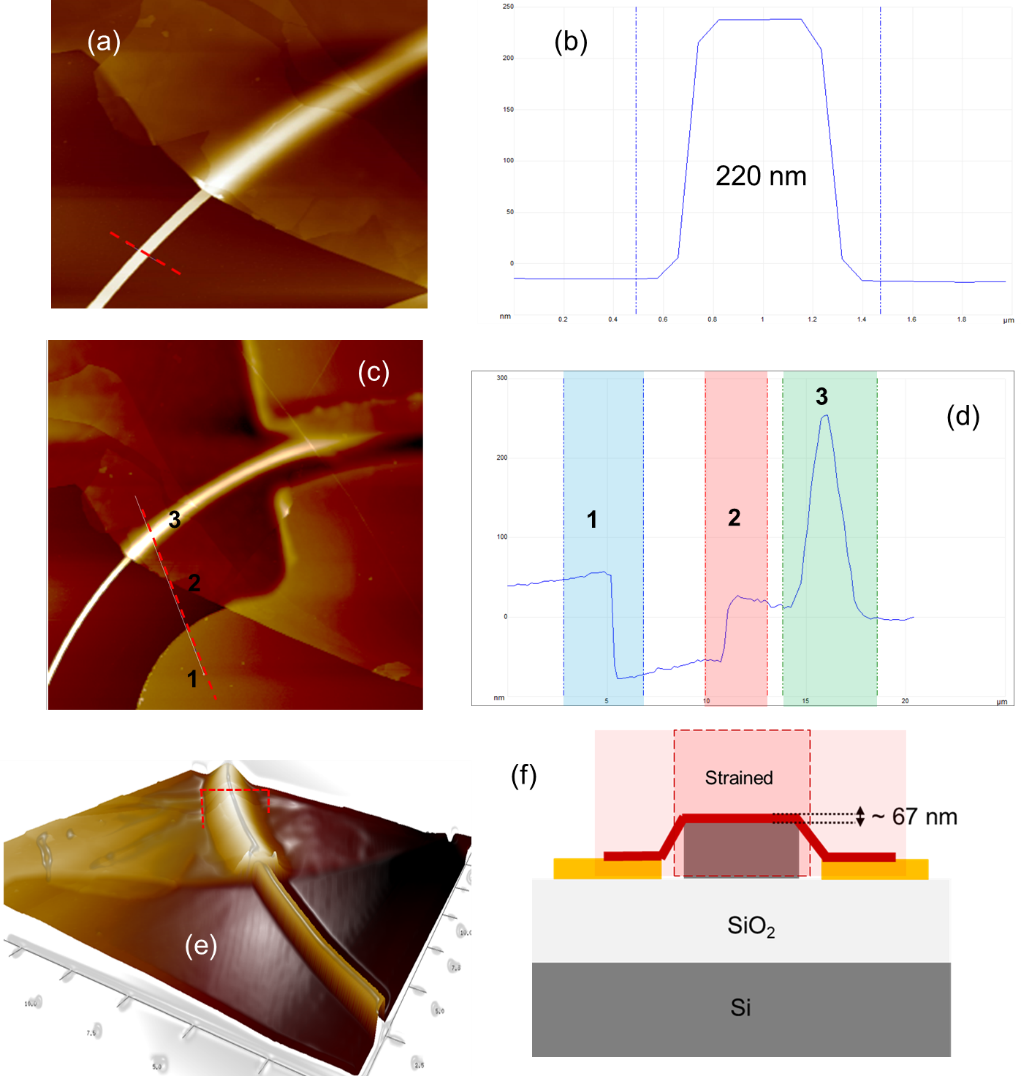


**Figure S10.** (a) AFM scan of integrated CCPS on Si-MRR (b) cross section of the red dashed line in (a) showing the height of the striped waveguide (c) zoom in AFM scan of integrated CCPS on Si-MRR presented in Fig.S9a (d) cross-sectional view along the dashed red line in (c), illustrating the height variations across the device structure as 1, 2,3 regions (e) 3D reconstructed image derived from the AFM scan in (c), depicting the topographical detail of the CCPS on the MRR (f) schematic representation on the strain effect as 2D side view of the red dashed square in (e).

The presence of spatially varying strain in our device, a consequence of the non-planar waveguide structure. This strain is apparent in both the AFM cross-sectional view and the 3D reconstructed image. As indicated in Reference 52 of our manuscript, such local tensile strain can lead to a graded bandgap, potentially increasing the material's overall absorption by diminishing the bandgap. However, in our investigation, while we acknowledge the strain induced by the 100 nm difference in height between the metal electrode and the Si waveguide, we have experimentally ascertained that this strain does not significantly influence the bandgap to the extent of affecting the material's optical loss or its passive nature. Our material has a bandgap of 1.35 eV, and a considerable reduction to 0.9 eV (a 500 meV effect due to strain) would be required to initiate optical loss at SWIR (1310 nm), a scenario not observed in our results.

**
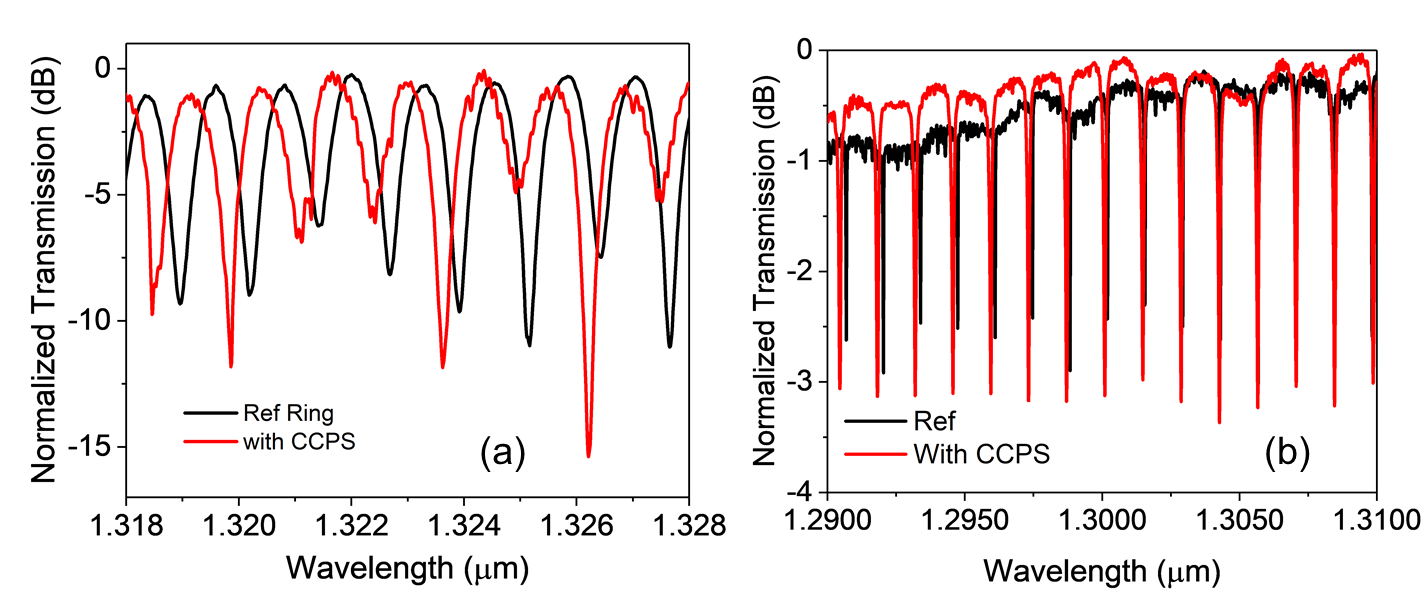
**

**Figure S11.** Optical transmission measurements (a) transverse magnetic polarization for ~ 65–67 nm and a coverage length of about 30 μm (b) transverse electric polarization for ~ 65–67 nm with 38 µm coverage length.

1. **Optical losses in CCPS using microring resonator**

The change in optical loss per length (Δα) of the ring caused by the multilayer CCPS in the evanescent region is extracted using the reduction observed in the ring’s Q-factor. The ring’s intrinsic quality factor Q is linked to the loss per unit length as follows^6,7^:

$Q=\frac{10}{ln(10)}\frac{2\pi n_{g}}{\lambda_{0}\alpha}$ (1)

Where λ_0_ is the resonance wavelength and n_g_ is the group index.

As illustrated in Fig. 7b in the main manuscript, the loaded quality factor (λ_0_/FWHM) is extracted from the experimental transmission spectra using Lorentz fitting.

The intrinsic Q in Eq 1 can be calculated from the loaded Q values as follows^8^:

$Q_{i=}\frac{{2Q}_{l}}{1\pm\sqrt{T_{0}}}$ (2)

In which Q_i_ is intrinsic quality factor, Q_l_ is loaded Q, and T_0_ is the normalized transmitted power at the resonance wavelength.

Our chips design is optimized for critical coupling cavity condition at 1310 nm, hence an intrinsic quality factor is ~ 2Q_l_

After CCPS integration, the losses primarily originate from reflections and scattering at the coupling interface between the passive waveguide (air/Si) and the hybrid region, involving mode mismatching losses and flake irregularities. Negligible losses are attributed to material absorption in the SWIR region. The change in the optical loss Δα = α_f_-α_i_ can be expressed as^6^:

$$\Delta\alpha=\frac{2\pi n_{g}}{\lambda_{0}}\frac{10}{\ln(10)}\left( \frac{1}{Q_{f}}-\frac{1}{Q_{i}} \right) (3)$$

**Note**: 10/ln (10) is the conversion from linear to logarithmic.

Here, Q_f_ is the composite ring quality factor (after integrating CCPS) and Q_i_ is the bare ring’s quality factor (before integration).

The n_g_ (4.18) value was extracted from the lumerical simulation, the quality factor of the ring before and after was calculated from the measured FWHM of the resonators transmission spectra as represented in Fig.7b using Lorentz fitting. By substituting the measured values obtained for the quality factor before and after integration at a resonant wavelength of 1.30706 µm in equation 3 and using equation 2 to obtain the intrinsic Q, the optical loss due to CCPS can be calculated. This value was then normalized to the CCPS interaction length (2πR/L).

1. **Change in the effective index of refraction calculation**

We calculate the change in the real part of the effective index (Δ𝑛_eff_) of the propagating mode with varying voltage from the change in the resonance wavelength (Δλ) of the transmission response of a ring resonator critically coupled to a bus waveguide (see figure S2) using ^9^:

$$\Delta n_{eff}=\frac{\lambda_{0}\Delta\lambda}{FSR*L}$$

where λ_0_ is the resonance wavelength at 0 V bias, FSR is the free-spectral range (1.4 nm) in terms of wavelength, and L (20 µm) is the CCPS interaction length. **Note:** we calculate the experimental Δ𝑛 by assuming that the phase modulation is entirely due to the Cu migration in CCPS and hence the Δ𝑛 of CCPS equals the Δn_eff_ of propagating mode.

**Electro-optic testing setup**

To measure the fabricated device, we use as an input a NIR laser source (Tunable Laser Source Keysight 81606A-113) that is tunable from 1.28 to 1.36 μm and has a 10 kHz linewidth. After sending the beam through a polarization controller, we couple light into the chip through a lensed fiber (side coupling to the chip). The output response from the devices is collected by an output lensed fiber and detected by an optical power meter. A source meter was used to control the biases and measure the I-V characteristics via a pair of standard DC electrical probes under dark conditions.


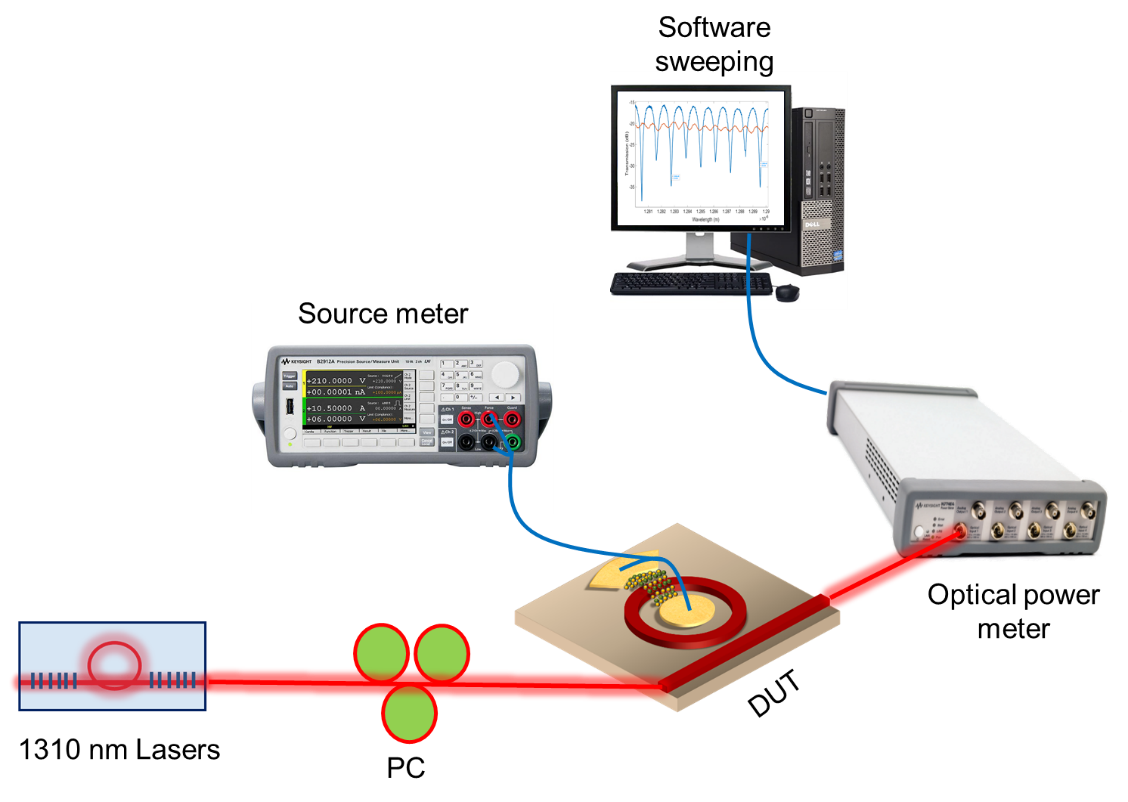


**Figure S12.** Experimental setup of electro-optic tuning of CCPS/Si MRR.

1. **Underlying mechanism of refractive index change**

When an electric field is applied to the ferroionic material, Cu ions migrate, leading to an accumulation at one electrode while depleting at the other. This movement effectively creates regions within the material that mimic p-type and n-type semiconductor behavior. The electrode with Cu ion accumulation exhibits n-type characteristics, whereas the electrode with Cu ion depletion exhibits p-type characteristics. This redistribution of Cu ions and the resultant creation of p-type and n-type regions alter the local electronic environment, thus affecting the material's refractive index and leading to a blue shift in the optical resonance. Ion migration's impact on the refractive index of materials has been documented in other systems^10^.

This behavior parallels the phenomena observed in phase shifters based on silicon and TMDs (MoS_2_ and WS_2_) operating in capacitive mode^11^. In silicon and TMDs, carrier accumulation (electrons in n-type and holes in p-type regions) or inversion leads to a change in the refractive index, thus resulting in a unidirectional shift. While the charge carriers in these materials are electrons and holes, in our ferroionic material, they are Cu ions. Nevertheless, the underlying principle remains that the redistribution of charge carriers, be it Cu ions in our material or electrons and holes in silicon, affects the refractive index in a similar manner.

The observation of a consistent blue shift, regardless of the polarity of the applied voltage, is thus explained by the symmetric nature of this ion migration. Whether the Cu ions migrate left or right, the change in ion concentration on either side of the material leads to an analogous modification in the local electronic structure, manifesting as a blue shift in the optical resonance.


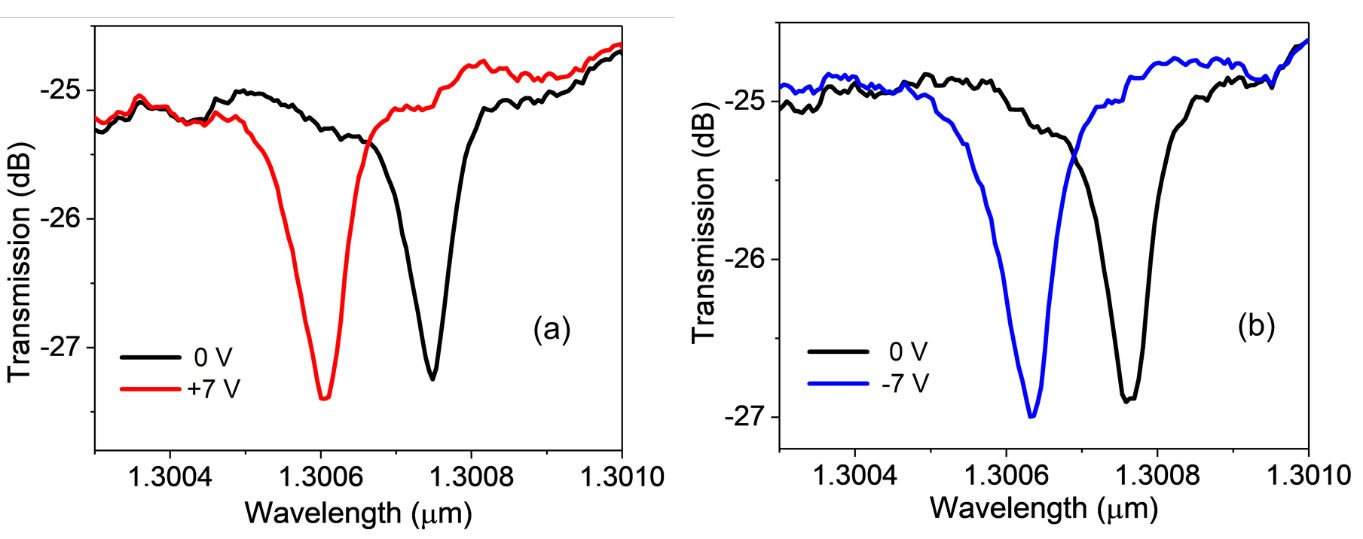


**Figure S13.** The transmission spectra of TE mode of composite MRR at (a) 0 V and +7 V

(b) 0 V and -7 V.


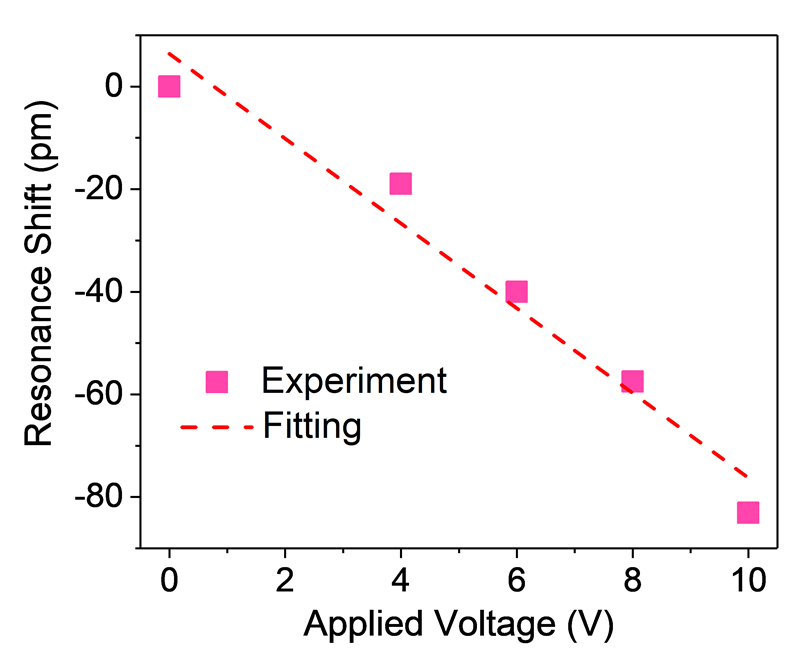


**Figure S14.** The resonance shift as a function of applied voltage.


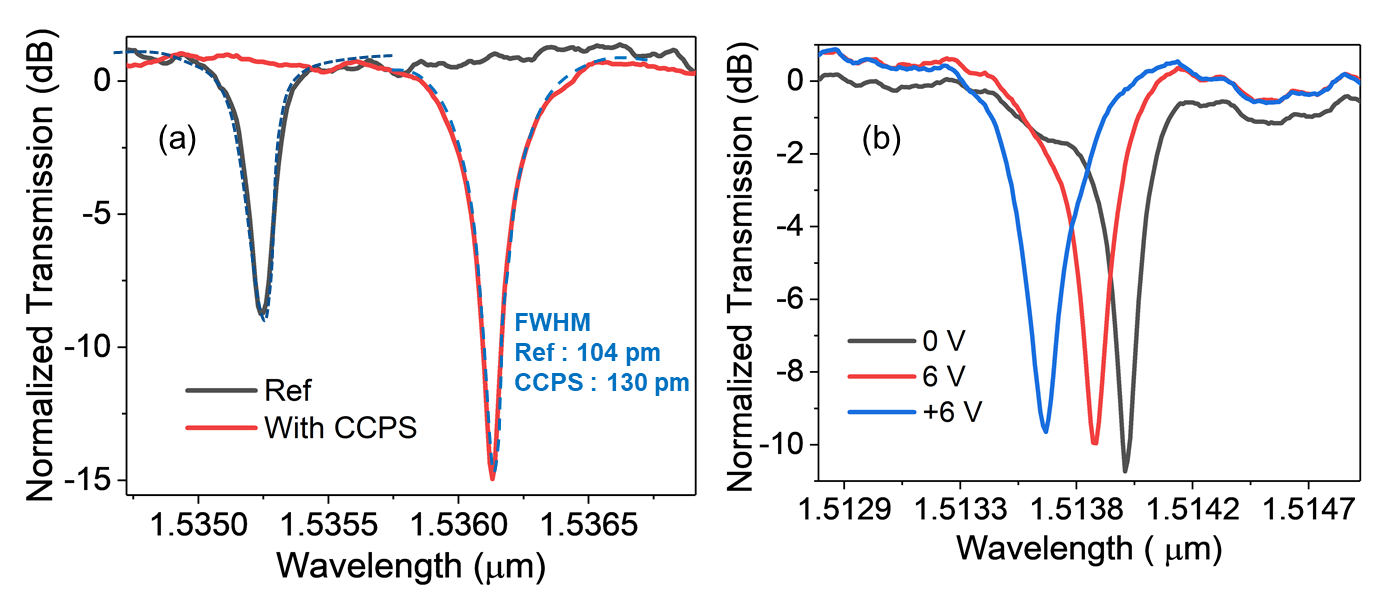


**Figure S15.** (a) The resonance dips observed in the MRR both with and without the integration of CCPS. The dashed line represents the Lorentz fitting, while the solid line shows the actual experimental transmission spectra (b) The transmission spectra of the MRR recorded at a steady voltage of 6 V with a polling time represented as +6 V, specifically for transverse electric light polarization at a wavelength of 1550 nm.

1. **Comparative analysis and device testing**

Our fabricated devices were constructed using two photonic chips, each includes 15 ring resonators. Figure S16a illustrates one of these chips prior to the transfer process (pristine chip). We developed a specialized in-house transfer technique to integrate 2D materials into the photonic chips (see Fig.16b). Initially, CCPS flakes were mechanically exfoliated using Nitto SPV224 PVC tape, then transferred to a PDMS film. A gold-plated needle-based micro stamper, positioned using a micro-positioner, facilitated the transfer of the material from the PDMS. This transparent PDMS, alignable under an optical microscope, allowed for precise placement of the flakes on the photonic chip. By scanning the PDMS under a microscope, suitable flakes were identified and selected for transfer, ensuring no cross-contamination and proper geometry. The stamp, with a contact area larger than the flake, precisely positioned the flake onto the target device. This method enabled us to transfer several devices, achieving uniform CCPS layers on the rings (identifiable by consistent flake color contrast) with interaction lengths between 4 and 20 µm. Figure 17a presents the SEM images of the various transferred devices used in our study.


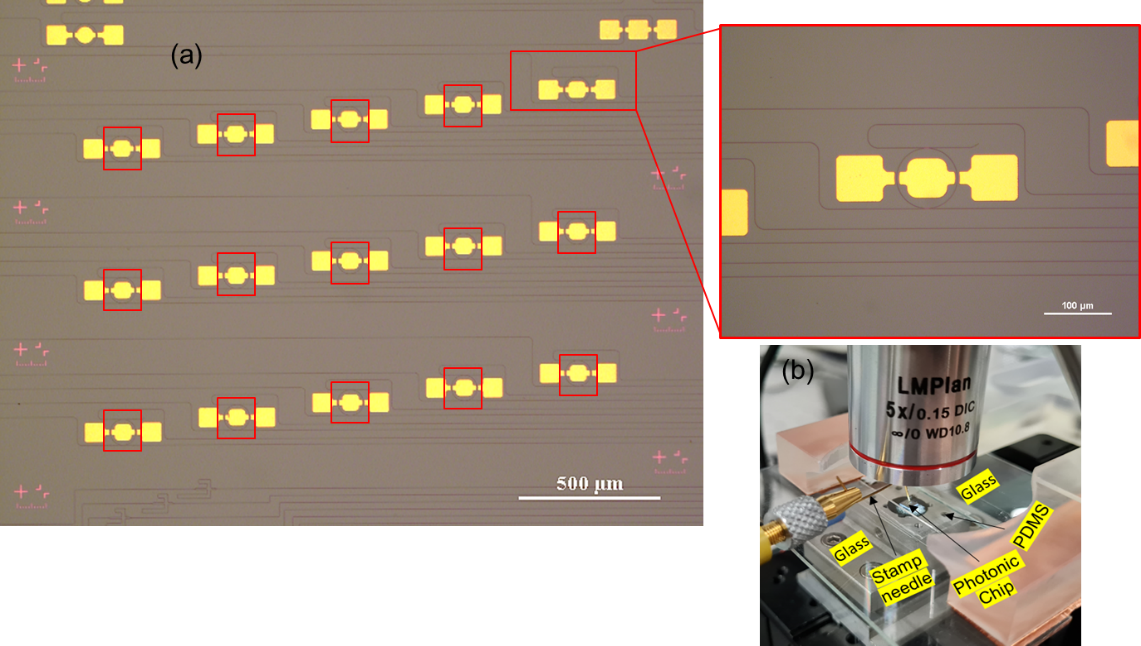


**Figure S16.** (a) Optical microscopy image of the photonic chip as fabricated, prior to the transfer of CCPS. It features 15 ring resonator devices, each delineated by red squares, on the chip. Additionally, zoom-in view of one of the ring resonators, as employed in our devices (b) in-house deterministic dry transfer setup.

We carried out a further comparative analysis to demonstrate the variation in the effective refractive index across devices, contingent upon the length of flake coverage. The defining factor for the maximum active region in these devices is the length of the gold electrode. Consequently, devices in which the flake spans the entire electrode length (20 µm) typically exhibit the greatest change in the refractive index, with values reaching up to 3.3 x 10^-3^. In contrast, devices with less flake coverage, identifiable by the red dashed lines in the Scanning Electron Microscopy (SEM) images, exhibit a smaller change in the effective refractive index (see Fig.S17 b). This analysis underscores the influence of flake coverage on the refractive index modulation of the devices. During our measurements we also observed that devices with the same coverage (20 µm) exhibited variations in Δn_eff_, ranging from 2 x10^-3^ RIU to 3.3 x10^-3^ RIU. These differences could be attributed to the material's electrical anisotropy stemming from varying crystal orientations^12^.

**
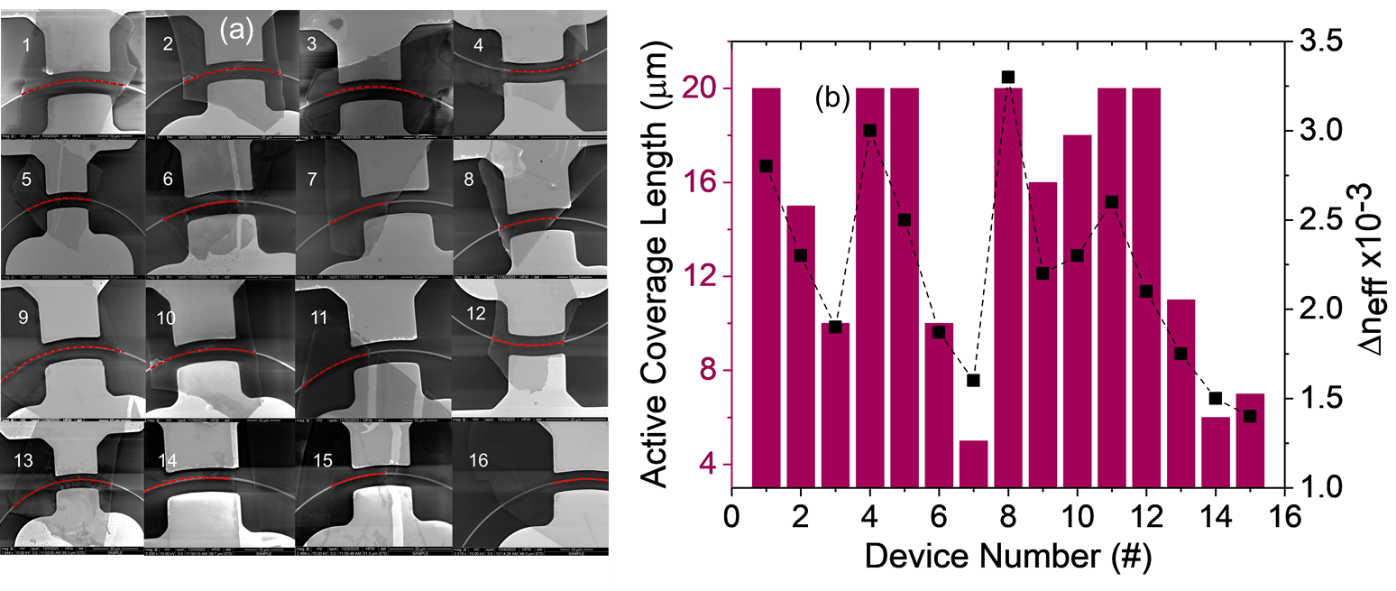
**

**Figure S17.** (a) Scanning electron microscopy images of transferred and tested devices during our experiments (b) coverage length and change in effective refractive index of the 15 tested devices.

**References**

1. Jiang, X. *et al.* Manipulation of current rectification in van der Waals ferroionic CuInP_2_S_6_. *Nat. Commun.* **13**, 574 (2022).

2. Zotev, P. G. *et al.* Van der Waals Materials for Applications in Nanophotonics. *Laser Photonics Rev.* **17**, 2200957 (2023).

3. Dushaq, G., Villegas, J. E., Paredes, B., Tamalampudi, S. R. & Rasras, M. S. Anisotropic Van Der Waals 2D GeAs Integrated on Silicon Four-Waveguide Crossing. *J. Light. Technol.* **41**, 1784–1789 (2023).

4. Dushaq, G., Paredes, B., Villegas, J. E., Tamalampudi, S. R. & Rasras, M. On-chip integration of 2D Van der Waals germanium phosphide (GeP) for active silicon photonics devices. *Opt. Express* **30**, 15986 (2022).

5. Tamalampudi, S. R., Dushaq, G., Villegas, J. E., Paredes, B. & Rasras, M. S. A Multi-layered GaGeTe Electro-Optic Device Integrated in Silicon Photonics. *J. Light. Technol.* 1–7 (2023) doi:10.1109/JLT.2023.3237818.

6. Wei, G., Stanev, T. K., Czaplewski, D. A., Jung, I. W. & Stern, N. P. Silicon-nitride photonic circuits interfaced with monolayer MoS2. *Appl. Phys. Lett.* **107**, 091112 (2015).

7. Rabiei, P., Steier, W. H., Cheng Zhang & Dalton, L. R. Polymer micro-ring filters and modulators. *J. Light. Technol.* **20**, 1968–1975 (2002).

8. Miller, S. A. *et al.* Low-loss silicon platform for broadband mid-infrared photonics. *Optica* **4**, 707 (2017).

9. Ortmann, J. E. *et al.* Ultra-Low-Power Tuning in Hybrid Barium Titanate–Silicon Nitride Electro-optic Devices on Silicon. *ACS Photonics* **6**, 2677–2684 (2019).

10. Toney Fernandez, T. *et al.* Ion migration assisted inscription of high refractive index contrast waveguides by femtosecond laser pulses in phosphate glass. *Opt. Lett.* **38**, 5248 (2013).

11. Chen, H. *et al.* Heterogeneous integrated phase modulator based on two-dimensional layered materials. *Photonics Res.* **10**, 1401–1407 (2022).

12. Wang, X. *et al.* Electrical and magnetic anisotropies in van der Waals multiferroic CuCrP2S6. *Nat. Commun.* **14**, 840 (2023).
